# A new non-monotonic infeasible simplex-type algorithm for Linear Programming

**DOI:** 10.7717/peerj-cs.265

**Published:** 2020-03-30

**Authors:** Charalampos P. Triantafyllidis, Nikolaos Samaras

**Affiliations:** 1 Computational Biology & Integrative Genomics, Department of Oncology, Medical Sciences Division, University of Oxford, Oxford, United Kingdom; 2 Department of Applied Informatics, School of Information Sciences, University of Macedonia, Thessaloniki, Greece

**Keywords:** Linear programming, Simplex-type, Interior point method, Exterior point, Non-monotonic, Infeasible, Mathematical programming, Optimization

## Abstract

This paper presents a new simplex-type algorithm for Linear Programming with the following two main characteristics: (i) the algorithm computes basic solutions which are neither primal or dual feasible, nor monotonically improving and (ii) the sequence of these basic solutions is connected with a sequence of monotonically improving interior points to construct a feasible direction at each iteration. We compare the proposed algorithm with the state-of-the-art commercial CPLEX and Gurobi Primal-Simplex optimizers on a collection of 93 well known benchmarks. The results are promising, showing that the new algorithm competes versus the state-of-the-art solvers in the total number of iterations required to converge.

## Introduction

Linear Programming (LP) constitutes one of the most fundamental classes of mathematical programming models which is widely used in many scientific areas since many real world problems can be formulated as Linear Programs (LPs) (Triantafyllidis & Papageorgiou, 2018; Gkioulekas & Papageorgiou, 2019; Yang et al., 2016; Amin & Emrouznejad, 2011; Janssens & Ramaekers, 2011; Fernndez & Borrajo, 2012; Burdett et al., 2017). LP is an important tool nowadays in many applications, spanning across a broad spectrum of fields (Bertsimas & Tsitsiklis, 1997). Many algorithms have been invented for the solution of LPs. The majority of these algorithms can be divided into two main categories: (i) simplex-type or pivoting algorithms and (ii) Interior Point Methods (IPMs).

The Primal Simplex Algorithm (PSA) (Dantzig, 1949) had been the most efficient method for solving LPs until the 80’s. PSA ranked as one of the top 10 algorithms of the 20th century (Dongarra & Sullivan, 2000). It performs well in practice, particularly on LPs of small or medium size. Nevertheless, PSA is not polynomial. Its worst-case complexity is exponential (Klee & Minty, 1972). Simplex algorithm visits in a sequential manner adjacent vertices of the feasible region using pivot operations, so that the new vertex has better objective value (monotonic algorithm) compared to the previous one. It is well known that the behavior of this algorithmic family can be improved by modifying: (i) the initial solution and (ii) the pivoting rule. The selection of appropriate pivoting rules affects the number of iterations required for solving LPs. Different pivoting strategies yield different basis sequences in simplex-type algorithms. The flexibility of the entering and leaving variable selection allows to develop various pivoting schemes. A complete presentation can be found in Terlaky & Zhang (1993).

The first polynomial time algorithm for linear programming was the Russian (ellipsoid) algorithm, developed by Khachiyan (1979). However, the ellipsoid algorithm is impractical for LP. Karmarkar (1984) then invented the first Interior Point Method (IPM); it uses a sequence of interior points and converges to the optimal solution in a few number of iterations. The most important advantage of IPMs compared to PSA is that the number of iterations is not proportional or related in any manner to the number of vertices. Most of the IPMs are infeasible in nature (Mehrotra, 1992; Mehrotra, 1993) and it is broadly accepted that an infeasible primal-dual IPM is the most efficient algorithm of this family. The development of IPMs has revolutionized the field of mathematical programming and efficient IPMs outperform the PSA on large-scale LPs.

Despite this fact, LPs have continued to receive great scientific analysis lately. More and effective pivoting schemes appeared in the literature (Terlaky, 1985; Murty & Fathi, 1984; Arsham, 2007; Pan, 2008; Jurik, 2008; Yeh & Corley, 2009; Elhallaoui et al., 2011; Li, 2013). Additionally, the papers (Basu, Loera & Junod, 2014; Gleixner, Steffy & Wolter, 2016; Omer et al., 2015) proposed a framework for an improved Primal Simplex algorithm that guarantees an improvement in the objective value after each iteration. Also, during the last decades researchers proposed more efficient implementations of simplex-type algorithms.

The Exterior Point Simplex Algorithm (EPSA) was originally developed by Paparrizos (1991) for the assignment problem. EPSA can avoid the boundary of the polyhedron of the feasible region and constructs two paths to converge to the optimal solution. One path is exterior to the feasible region while the other one is feasible. Later on, Paparrizos (1993) generalized EPSA to LP. The key idea behind EPSA is that when using pivots based on feasible directions to select the pair of entering and leaving variables, the algorithm can converge faster to the optimal solution. Paparrizos, Samaras & Tsiplidis (2001) have demonstrated that the geometry of EPSA reveals that this algorithm is faster than PSA. This result was partially verified by preliminary computational results (Paparrizos, Samaras & Stephanides, 2003a; Paparrizos, Samaras & Triantafyllidis, 2008). A well established way to improve EPSA is to transform its exterior path into a dual feasible simplex path. Such an algorithm is called Primal-Dual Exterior Point Simplex Algorithm (PDEPSA) (Paparrizos, 1996). This algorithm requires an initial dual feasible basic solution. Since such a solution is not always readily available, a modified big-M method is applied. Variations of using a Two-Phase approach for the EPSA were presented in Triantafyllidis & Samaras (2014). The main advantage of PDEPSA is its promising computational performance.

An important improvement of the PDEPSA is to traverse across the interior of the feasible region, in an attempt to avoid degenerate vertices of vertex-following algorithms. This algorithm is called Primal-Dual Interior Point Simplex Algorithm (PDIPSA) (Samaras, 2001). PDIPSA can be seen as a separate procedure to move from any interior point to an optimal basic solution. It can be combined with IPMs in order to develop a hybrid algorithm consisting of two stages (Glavelis, Ploskas & Samaras, 2018). At first stage, an IPM is applied and at the second stage PDIPSA is applied to compute an optimal basic solution. The main advantage of this hybrid algorithm is that it exploits the strengths of both IPM and PDIPSA. The computational results are very encouraging. A complete review of Exterior Point algorithms can be found in Paparrizos, Samaras & Sifaleras (2015). A review paper summarizing the advantages and disadvantages of pivots, ellipsoid and IPMs was presented by Illes & Terlaky (2002). Several methods have been developed which provide a combination of IPMs with pivoting algorithms (Bixby et al., 1992; Bixby & Saltzman, 1994; Andersen & Ye, 1996; Pan, 2013).

All the above mentioned algorithms are monotonic in nature. A monotonic linear optimization algorithm starts with a (feasible or infeasible) vertex, moves between (adjacent or not) vertices, improving the value of the objective function until an optimal solution is found. In this paper a non-monotonic infeasible simplex-type algorithm for general LP is presented. The proposed method does not maintain monotonicity on the basic solutions, but only on the interior point which is used to construct the feasible direction at each iteration. This new algorithm is comprised of three different parts: (i) interior Exterior Primal Simplex Algorithm (iEPSA), (ii) Exterior Point Simplex Algorithm (EPSA) and (iii) Primal-Dual Interior Point Simplex Algorithm (PDIPSA). The first one (iEPSA) interconnects a primal interior point with a primal (infeasible) exterior one. Using these two points, a feasible direction is constructed and while iterating in a non-monotonic way the algorithm stops at either a primal or a dual feasible solution. On the other hand iEPSA improves strictly from iteration to iteration the objective value at the interior point. The exterior point reaches optimality independently of the monotonicity of the interior point. In conclusion, we have non-monotonic movement outside the feasible region and monotonic movement in the interior of the feasible region.

In order to gain insight into the practical behavior of the proposed algorithm, we have performed some computational experiments on a set of benchmark problems (netlib, Kennington, Mészáros). The computational results demonstrate that the proposed non-monotonic algorithm requires less iterations than both the Primal-Simplex algorithm implemented in CPLEX and Gurobi commercial solvers.

This paper is organized as follows: In ‘Materials & Methods’ a brief reference to some basic notation for the linear problem and the algorithms described in this paper is given. Subsection *iEPSA* presents the proposed algorithm, an illustrative example and its pseudo-code. In the ‘Proof of Correctness’ subsection, mathematical proofs for the correctness of the algorithm are given. In order to gain an insight into the practical behavior of the proposed algorithm, we performed a computational study. These results are presented in the ‘Results’ section, followed by the ‘Conclusions’ section.

## Materials & Methods

In this section we give some necessary notation and definitions on LPs. Consider the following linear program in the standard form:

(1) \begin{eqnarray*}\begin{array}{@{}ll@{}} \displaystyle min&\displaystyle {c}^{T}x\\ \displaystyle subject to&\displaystyle Ax=b,\\ \displaystyle &\displaystyle x\geq 0 \end{array}\end{eqnarray*}

where *A* ∈ *R*^*m*×*n*^, (*c*, *x*) ∈ *R*^*n*^, *b* ∈ *R*^*m*^ and T denotes transposition. We assume that A has full rank, *rank*(*A*) = *m*, 1 ≤ *m* ≤ *n*. If *x* satisfies *Ax* = *b*, *x* ≥ 0, then *x* is a feasible solution. The dual problem associated with the Eq. (1) is presented in Eq. (2):

(2) \begin{eqnarray*}\begin{array}{@{}ll@{}} \displaystyle max&\displaystyle {b}^{T}y\\ \displaystyle subject to&\displaystyle {A}^{T}y+s=c,\\ \displaystyle &\displaystyle s\geq 0 \end{array}\end{eqnarray*}

where *y* ∈ *R*^*m*^ and *s* ∈ *R*^*n*^. Using a basic partition (*B*, *N*) of *A* as *A* = [*A*_*B*_*A*_*N*_] and the corresponding partitioning of *x*^*T*^ = [*x*_*B*_*x*_*N*_], *c*^*T*^ = [*c*_*B*_*c*_*N*_], Eq. (1) is written as:

(3) \begin{eqnarray*}\begin{array}{@{}ll@{}} \displaystyle min Z={c}_{B}^{T}{x}_{B}+{c}_{N}^{T}{x}_{N}&\displaystyle \\ \displaystyle subject to&\displaystyle \chskip[-4pc]{A}_{B}{x}_{B}+{A}_{N}{x}_{N}=b\\ \displaystyle &\displaystyle {x}_{B},{x}_{N}\geq 0 \end{array}\end{eqnarray*}

In Eq. (3), *A*_*B*_ is an *m* × *m* non-singular sub-matrix of *A*, called basic matrix or basis, whereas *A*_*N*_ is an *m* × (*n* − *m*) sub-matrix of *A* called non-basic matrix. The columns of *A* which belong to subset *B* are called basic and those which belong to *N* are called non-basic. The solution *x*_*B*_ = (*A*_*B*_)^−1^*b*, *x*_*N*_ = 0 is called a basic solution. The solution of Eq. (2) is computed by the relation *s* = *c* − *A*^*T*^*y*, where *y*^*T*^ = (*c*_*B*_)^*T*^(*A*_*B*_)^−1^ are the dual variables and *s* are the dual slack variables. The basis *A*_*B*_ is dual feasible iff *s* ≥ 0. The *i*th row of the coefficient matrix *A* is denoted by *A*_*i*._ and the *j*th column by *A*_.*j*_. Notation *x*_*B*[*i*]_ refers to the *i*th basic variable (*i*th element of vector *x*_*B*_). In solving LPs by pivoting methods, a huge amount of computational effort is consumed on the inversion of the basis *A*_*B*_. The basis is maintained in some factorized form. We use the LU-factorization available in MATLAB to compute the inverse of the basis in all three algorithms, iEPSA, EPSA and PDIPSA.

### The iEPSA method

A common characteristic of the majority of simplex-type algorithms is that they can be described as a process that uses simplex paths which lead to optimal solution. One advantage of the Exterior Point algorithms is that they use two paths to reach the optimal basis. One is feasible and the other infeasible (exterior). The relaxation of the feasibility constraints seems to be efficient in practice. Another potential advantage of EPSA is that should the initial direction be feasible (it spans the feasible region), the method can be applied directly on the original problem, without having to first compute an initial feasible basic solution thus completely avoiding Phase I. This is because EPSA never loses contact with the feasible region if the initial direction crosses it. On the other hand, one of the main disadvantages of the EPSA is the difficulty of constructing a *good* moving direction.

This drawback can be avoided if the exterior path is replaced with a dual feasible simplex path. It has been shown that by replacing the exterior path of an EPSA with a dual feasible simplex path results in an algorithm free from the computational disadvantages of EPSA (Paparrizos, Samaras & Stephanides, 2003b). A more effective version is the PDIPSA (Samaras, 2001). This algorithm can circumvent the problems of stalling and cycling more effectively and as a result improves the performance of the primal-dual exterior point algorithms. The advantage of PDIPSA emanates from the fact that it uses an interior point.

The iEPSA method is initialized with a pair of initial points: an infeasible basic solution and an interior point. The initial interior point (*x*_*interior*_) can be computed by applying an IPM in Eq. (1) with *c*^*T*^ = 0. Next, it constructs a feasible direction (*d* = *x*_*interior*_ − *x*_*current*_) and computes the pair of entering/leaving variables and a new (better) interior point. The above computations continue, swapping infeasible basic solutions on the exterior of the feasible region in a non-monotonic way, and in the interior by using better interior points (monotonic movement) in order to construct the search directions. The proposed method prioritizes monotonic pivots; however, should there be no monotonic eligible steps, the method moves to the least-worse non-monotonic infeasible basic solution.

If iEPSA finds a primal feasible basic solution, then the EPSA is applied to monotonically converge to the optimal solution. If at any given iteration iEPSA moves to a dual feasible partition then PDIPSA is applied. With the last interior point and the dual feasible partition from iEPSA, PDIPSA can also monotonically find the optimal solution.

#### Step-by-step description of iEPSA and pseudocode

The algorithm consists of two phases. In the first phase, the algorithm generates a sequence of points $({x}_{interior}^{i},{x}_{exterior}^{i})$, *i* = 0, 1, 2, ..., *T*, where ${x}_{interior}^{i}$ is a point in the relative interior of the feasible region for *i* = 0, ..., *T*, and ${x}_{exterior}^{i}$ is a basic solution to LP, that is infeasible to both the primal and the dual problem for *i* = 0, ..., *T* − 1. The first phase ends with a pair $({x}_{interior}^{T},{x}_{exterior}^{T})$, where the exterior point is either feasible to the primal or the dual problem. If the first phase ands with a basic feasible solution to the primal, then the second phase runs an algorithm called Exterior Point Simplex Algorithm (EPSA) from previous literature (Paparrizos, 1993), to obtain the optimal basic feasible solution. If the first phase ends with a dual feasible solution, the second phase runs an algorithm called Primal-Dual Interior Point Simplex Algorithm (PDIPSA), also from previous literature (Samaras, 2001). We show that the first phase method always ends with a basic solution that is feasible to either the primal or the dual problem. Thus, using the prior results on EPSA and PDIPSA, the overall algorithm is shown to correctly solve LP.

The main idea behind the first phase is the following: the algorithm is initialized with any basic (infeasible) solution ${x}_{exterior}^{0}$ and an interior point ${x}_{interior}^{0}$, found by a standard Interior Point Solver (in this case MOSEK IPM). At every iteration, *i* = 0, 1, 2, ... one computes the intersection of the line passing

 
 
 
 
 
1  Data: Eq. (1), Infeasible Basic Partition [B N], Interior Point xinterior 
2  Result: Primal or Dual feasible basic partition [__B __N] 
   _______________________________________________________________________________________________________________________________________________________________ 
  3  (Initialization) Compute: 
 4 (AB)−1,xB,wT, (sN)T 
 5 xcurrent = [ xB  xN] 
 6 d = xinterior − xcurrent 
 7 P = {j ∈ N : sj < 0}, Q = {j ∈ N : sj ≥ 0} 
 8 (sP )T = (cP )T − wTAP , (sQ)T = (cQ)T − wTAQ 
 9 (General loop) 
10  while xB(⁄≥ 0) do 
11       αα = xcurrent + αd : α =  xB[ra] _−dB[ra]  = min{ xB[i] _−dB[i]  : dB[i] < 0} ,∀i = 1,...,m 
12       ββ = xcurrent + βd : β =  xB[rb] _−dB[rb]  = max{ xB[i] _−dB[i]  : xB[i] < 0} ,∀i = 1,...,m 
13       if α = +∞ then 
14                   STOP-Eq. 1 is unbounded. 
         else 
15                   Find xmiddle = αα+ββ    2 
16                   if cTxmiddle < cTxinterior then 
17                      __xinterior = xmiddle 
                     else 
18                       if cTxmiddle = cTxinterior then 
19                            α = min{(xinterior)[i] c[i]         : −c[i] < 0} 
20                            __xinterior = xinterior + α 2 (−cT ) 
                         else 
21                            d = xinterior − xmiddle 
22                            α = min{(xinterior)[i] −(d)[i]      : (d)[i] < 0} 
23                            __xinterior = xinterior + α 2 d 
                         end if 
                     end if 
24                   Compute: 
25                   xB[rb] = xk 
26                   HrP = ((AB)−1)rb.AP 
27                   HrQ = ((AB)−1)rb.AQ, 
28                   θ1 = −sp Hrp = min{−sj Hrj : Hrj < 0,j ∈ P} 
29                   θ2 = −sq Hrq = min{−sj Hrj : Hrj < 0,j ∈ Q} 
30                   (Pivot-Update) 
31                   Find t1,t2 : P(t1) = p , Q(t2) = q. 
32                   if θ1 ≤ θ2 then 
33                                l = p. 
                     else 
34                                l = q 
                     end if  
35                   Find t : N(t) = l. Set N(t) = k , B(rb) = l . Update: 
36                   (AB)−1, xB,wT, (sN)T, (sP )T, (sQ)T , P,Q, __xcurrent = [ xB  xN] , __d = __xinterior −_xcurrent 
         end if 
    end while 
                                                 Algorithm 1: iEPSA    

through ${x}_{exterior}^{i}$ and ${x}_{interior}^{i}$ with the feasible region. This gives a line segment *l* (assuming the problem is bounded) with midpoint ${x}_{middle}^{i}$. Otherwise, one takes a *half-step* from the current ${x}_{interior}^{i}$ in the direction of ${x}_{middle}^{i}$ and sets this as the new ${x}_{interior}^{i+1}$. One also computes the endpoint of the line segment *l* closest to ${x}_{interior}^{i}$. This endpoint lies on some facet of the feasible region. This facet dictates which nonbasic variable will enter the basis and an appropriate exiting variable is selected. This then gives the new basic solution ${x}_{exterior}^{i+1}$.

**Figure 1:**
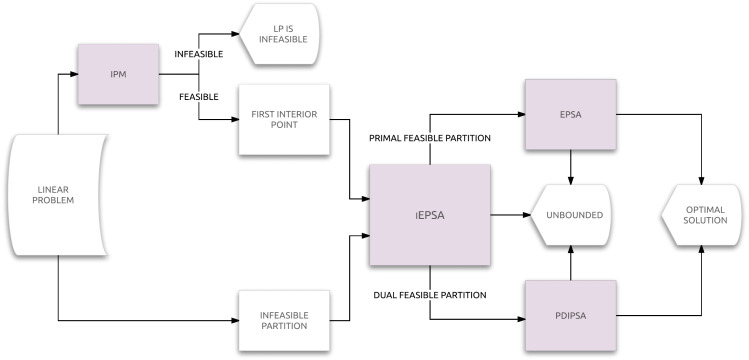
Flow diagram of iEPSA.

A flow diagram of iEPSA combined with EPSA and PDIPSA to provide an integrated solver for LP is shown in Fig. 1. A formal description of the iEPSA method is given in Algorithm 1.

#### An example

We will briefly demonstrate the iEPSA method in a simple example. Points {*αα*, *ββ*} represent the exiting and entering boundary points correspondingly for each feasible direction spanning the polyhedron. Assume we are given the following linear programming problem:

(4) \begin{eqnarray*}\begin{array}{@{}crcrcl@{}} \displaystyle \max \nolimits &\displaystyle Z={x}_{1}&\displaystyle +&\displaystyle {x}_{2}&\displaystyle &\displaystyle \\ \displaystyle \mathrm{subject~ to}&\displaystyle {x}_{1}&\displaystyle -&\displaystyle {x}_{2}&\displaystyle \leq &\displaystyle 2\\ \displaystyle &\displaystyle -{x}_{1}&\displaystyle +&\displaystyle {x}_{2}&\displaystyle \leq &\displaystyle 4\\ \displaystyle &\displaystyle 3{x}_{1}&\displaystyle +&\displaystyle 5{x}_{2}&\displaystyle \leq &\displaystyle 30\\ \displaystyle &\displaystyle -4{x}_{1}&\displaystyle -&\displaystyle 13{x}_{2}&\displaystyle \leq &\displaystyle -23\\ \displaystyle &\displaystyle {x}_{1}&\displaystyle -&\displaystyle 8{x}_{2}&\displaystyle \leq &\displaystyle -12\\ \displaystyle &\displaystyle 8{x}_{1}&\displaystyle -&\displaystyle 5{x}_{2}&\displaystyle \leq &\displaystyle 3\\ \displaystyle &\displaystyle &\displaystyle &\displaystyle {x}_{i}&\displaystyle \geq &\displaystyle 0,\forall i\in \left\{ 1,2 \right\} \end{array}\end{eqnarray*}

**Figure 2:**
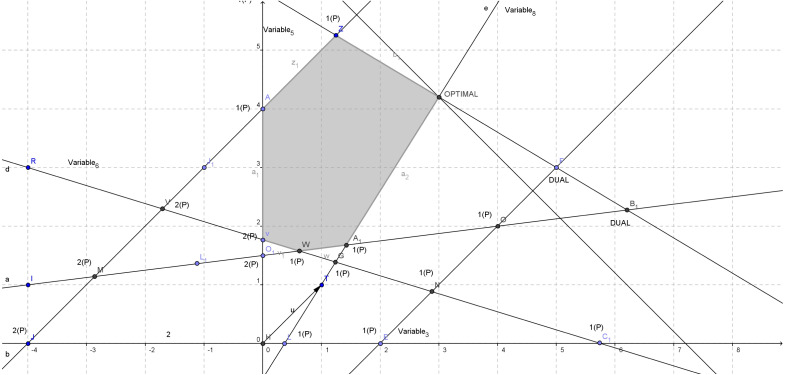
Feasible region and duality on vertexes for Eq. (4).

The corresponding feasible region is depicted in Fig. 2. There exist in total eight different variables after the addition of the slack ones. The axis system represents variables *x*_1_ (y=0) and *x*_2_ (x=0). The numbers with *P* in brackets on the right at each basic solution stand for the number of elements in vector *P*. The optimal point is [3,4.2] and the optimal objective value is *Z* = 7.2. After the addition of the slack variables we have:

\begin{eqnarray*}A= \left[ \begin{array}{@{}cccccccc@{}} \displaystyle 1&\displaystyle -1&\displaystyle 1&\displaystyle 0&\displaystyle 0&\displaystyle 0&\displaystyle 0&\displaystyle 0\\ \displaystyle -1&\displaystyle 1&\displaystyle 0&\displaystyle 1&\displaystyle 0&\displaystyle 0&\displaystyle 0&\displaystyle 0\\ \displaystyle 3&\displaystyle 5&\displaystyle 0&\displaystyle 0&\displaystyle 1&\displaystyle 0&\displaystyle 0&\displaystyle 0\\ \displaystyle -4&\displaystyle -13&\displaystyle 0&\displaystyle 0&\displaystyle 0&\displaystyle 1&\displaystyle 0&\displaystyle 0\\ \displaystyle 1&\displaystyle -8&\displaystyle 0&\displaystyle 0&\displaystyle 0&\displaystyle 0&\displaystyle 1&\displaystyle 0\\ \displaystyle 8&\displaystyle -5&\displaystyle 0&\displaystyle 0&\displaystyle 0&\displaystyle 0&\displaystyle 0&\displaystyle 1\\ \displaystyle \end{array} \right] ,c= \left[ \begin{array}{@{}c@{}} \displaystyle -1,-1,0,0,0,0,0,0\\ \displaystyle \end{array} \right] ,b= \left[ \begin{array}{@{}c@{}} \displaystyle 2\\ \displaystyle 4\\ \displaystyle 30\\ \displaystyle -23\\ \displaystyle -12\\ \displaystyle 3\\ \displaystyle \end{array} \right] \end{eqnarray*}

**Initialization**

Assume we start with the infeasible partition *B* = [1, 3, 4, 5, 7, 8], *N* = [2, 6] and the following interior point *x*_*interior*_ (calculated from MOSEK’s IPM). All the appropriate computations are:

\begin{eqnarray*}({A}_{B})^{-1}= \left[ \begin{array}{@{}cccccc@{}} \displaystyle 1&\displaystyle 0&\displaystyle 0&\displaystyle 0.25&\displaystyle 0&\displaystyle 0\\ \displaystyle 0&\displaystyle 1&\displaystyle 0&\displaystyle -0.25&\displaystyle 0&\displaystyle 0\\ \displaystyle 0&\displaystyle 0&\displaystyle 1&\displaystyle 0.75&\displaystyle 0&\displaystyle 0\\ \displaystyle 0&\displaystyle 0&\displaystyle 0&\displaystyle 0.25&\displaystyle 1&\displaystyle 0\\ \displaystyle 0&\displaystyle 0&\displaystyle 0&\displaystyle -0.25&\displaystyle 0&\displaystyle 0\\ \displaystyle 0&\displaystyle 0&\displaystyle 0&\displaystyle 2&\displaystyle 0&\displaystyle 1\\ \displaystyle \end{array} \right] ,({s}_{N})^{T}= \left[ \begin{array}{@{}c@{}} \displaystyle 2.25,-0.25 \end{array} \right] ,{w}^{T}= \left[ \begin{array}{@{}c@{}} \displaystyle 0,0,0,0.25,0,0 \end{array} \right] \end{eqnarray*}

\begin{eqnarray*}{x}_{B}= \left[ \begin{array}{@{}c@{}} \displaystyle -3.75\\ \displaystyle 9.75\\ \displaystyle 12.75\\ \displaystyle -17.75\\ \displaystyle 5.75\\ \displaystyle -43 \end{array} \right] ,{x}_{current}= \left[ \begin{array}{@{}c@{}} \displaystyle 5.75\\ \displaystyle 0\\ \displaystyle -3.75\\ \displaystyle 9.75\\ \displaystyle 12.75\\ \displaystyle 0\\ \displaystyle -17.75\\ \displaystyle -43 \end{array} \right] ,{x}_{interior}= \left[ \begin{array}{@{}c@{}} \displaystyle 0.3189\\ \displaystyle 3.0877\\ \displaystyle 4.7688\\ \displaystyle 1.2312\\ \displaystyle 13.6047\\ \displaystyle 18.4160\\ \displaystyle 12.3829\\ \displaystyle 15.8874 \end{array} \right] \end{eqnarray*}

Here *w* are the dual variables. The *N* set of indexes actually represents the current non-basic solution. In our case [2,6] is the 2-D point [5.75,0]. A feasible direction *d* is then constructed by connecting the interior point with the infeasible basic solution:

\begin{eqnarray*}d={x}_{interior}-{x}_{current}= \left[ \begin{array}{@{}c@{}} \displaystyle 0.3189\\ \displaystyle 3.0877\\ \displaystyle 4.7688\\ \displaystyle 1.2312\\ \displaystyle 13.6047\\ \displaystyle 18.4160\\ \displaystyle 12.3829\\ \displaystyle 15.8874 \end{array} \right] - \left[ \begin{array}{@{}c@{}} \displaystyle 5.75\\ \displaystyle 0\\ \displaystyle -3.75\\ \displaystyle 9.75\\ \displaystyle 12.75\\ \displaystyle 0\\ \displaystyle -17.75\\ \displaystyle -43 \end{array} \right] = \left[ \begin{array}{@{}c@{}} \displaystyle -5.4311\\ \displaystyle 3.0877\\ \displaystyle 8.5188\\ \displaystyle -8.5188\\ \displaystyle 0.8547\\ \displaystyle 18.4160\\ \displaystyle 30.1329\\ \displaystyle 58.8874 \end{array} \right] \end{eqnarray*}

Mapping now the direction *d* on the basic variables we get *d*_*B*_:

\begin{eqnarray*}{d}_{B}= \left[ \begin{array}{@{}c@{}} \displaystyle 8.5188\\ \displaystyle -8.5188\\ \displaystyle 0.8547\\ \displaystyle 30.1329\\ \displaystyle -5.4311\\ \displaystyle 58.8874 \end{array} \right] \end{eqnarray*}

**Figure 3:**
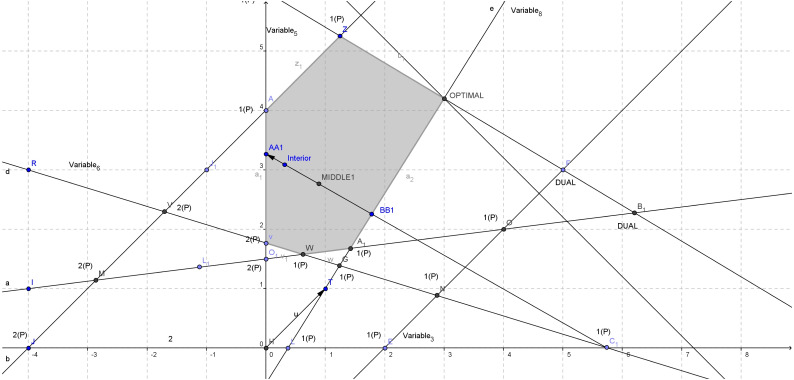
Constructing the first direction for Eq. (4).

Also we have *P* = [6] and *Q* = [2]. The direction from the initial infeasible basic solution (5.75,0) to the interior point is shown in Fig. 3. Since *x*_*current*_ is our initial infeasible basic solution and *d* is our current feasible direction, this direction intersects the feasible region at an exiting point *AA*1 (as shown in Fig. 3) which can be calculated using the relations below:

**General loop - Iteration 1**

\begin{eqnarray*}\alpha = \frac{{x}_{B[r]}}{-{d}_{B[r]}} =min \left\{ \frac{{x}_{B[i]}}{-{d}_{B[i]}} :{d}_{B[i]}\lt 0 \right\} & =min \left\{ \frac{{x}_{B}[2,5]}{-{d}_{B}[2,5]} \right\} =min \left\{ \frac{9.75}{8.5188} , \frac{5.75}{5.4311} \right\} =\nonumber\\\displaystyle min\{1.1445,1.0587\}=1.0587 \end{eqnarray*}

\begin{eqnarray*}\alpha \alpha ={x}_{current}+\alpha d= \left[ \begin{array}{@{}c@{}} \displaystyle 5.75\\ \displaystyle 0\\ \displaystyle -3.75\\ \displaystyle 9.75\\ \displaystyle 12.75\\ \displaystyle 0\\ \displaystyle -17.75\\ \displaystyle -43 \end{array} \right] +1.0587 \left[ \begin{array}{@{}c@{}} \displaystyle -5.4311\\ \displaystyle 3.0877\\ \displaystyle 8.5188\\ \displaystyle -8.5188\\ \displaystyle 0.8547\\ \displaystyle 18.4160\\ \displaystyle 30.1329\\ \displaystyle 58.8874 \end{array} \right] = \left[ \begin{array}{@{}c@{}} \displaystyle 0\\ \displaystyle 3.2690\\ \displaystyle 5.2690\\ \displaystyle 0.7310\\ \displaystyle 13.6549\\ \displaystyle 19.4973\\ \displaystyle 14.1522\\ \displaystyle 19.3451 \end{array} \right] \end{eqnarray*}

In a similar manner, the entering point *BB*1 (as shown in Fig. 3) can be calculated using a maximum ratio test:

\begin{eqnarray*}\beta = \frac{-{x}_{B[r]}}{{d}_{B[r]}} =max \left\{ \frac{-{x}_{B[i]}}{{d}_{B[i]}} :{x}_{B[i]}\lt 0 \right\} =max \left\{ \frac{-{x}_{B}[1,4,6]}{{d}_{B}[1,4,6]} \right\} =\nonumber\\\displaystyle max \left\{ \frac{-3.75}{-8.5188} , \frac{-17.75}{-30.1329} , \frac{-43}{-58.8874} \right\} =max\{0.4402,0.5891,0.7302\}=0.7302 \end{eqnarray*}

Hence, r_b_ = 3, then the 3^*rd*^ element of [1, 4, 6] is r = 6.

The entering point then is:

\begin{eqnarray*}\beta \beta ={x}_{current}+\beta d= \left[ \begin{array}{@{}c@{}} \displaystyle 5.75\\ \displaystyle 0\\ \displaystyle -3.75\\ \displaystyle 9.75\\ \displaystyle 12.75\\ \displaystyle 0\\ \displaystyle -17.75\\ \displaystyle -43 \end{array} \right] +0.7302 \left[ \begin{array}{@{}c@{}} \displaystyle -5.4311\\ \displaystyle 3.0877\\ \displaystyle 8.5188\\ \displaystyle -8.5188\\ \displaystyle 0.8547\\ \displaystyle 18.4160\\ \displaystyle 30.1329\\ \displaystyle 58.8874 \end{array} \right] = \left[ \begin{array}{@{}c@{}} \displaystyle 1.7842\\ \displaystyle 2.2547\\ \displaystyle 2.4705\\ \displaystyle 3.5295\\ \displaystyle 13.3741\\ \displaystyle 13.4475\\ \displaystyle 4.2533\\ \displaystyle 0 \end{array} \right] \end{eqnarray*}

It is easy now to compute the middle point *MIDDLE*1 (as shown in Fig. 3) between *αα* − *ββ*:

\begin{eqnarray*}{x}_{middle}= \frac{\alpha \alpha +\beta \beta }{2} = \left[ \begin{array}{@{}c@{}} \displaystyle 0.8921\\ \displaystyle 2.7618\\ \displaystyle 3.8697\\ \displaystyle 2.1303\\ \displaystyle 13.5145\\ \displaystyle 16.4724\\ \displaystyle 9.2027\\ \displaystyle 9.6726\\ \displaystyle \end{array} \right] \end{eqnarray*}

We observe the following:

• Both exiting and entering points have only one zero element which was expected since both points are boundary in a 2-D problem
• Maximum step size *β* is less than the minimum step *α* so as the entering point is closer to the infeasible basic solution and the exiting furthest as expected, since the direction will intersect the feasible region
• These two boundary points define a unique feasible ray segment from *ββ* to *αα* (*ββ* → *αα*)
• The objective function value at *ββ* is better than in *αα* (direction is non-improving) and the objective function value at the middle point is better than the initial interior point.

Since the middle point has better objective value than the initial interior point (it is: *Z*_*middle*_ = (*c*^*T*^*middle*) = [ − 0.8921,  − 2.7618] =  − 3.6539 and *Z*_*x*_*interior*__ = (*c*^*T*^*x*_*interior*_) = [ − 0.3189,  − 3.0877] =  − 3.4066) we keep this middle point as an improved interior point for the next iteration. We now choose the leaving variable according to *ββ* boundary point: *k* = 8, *r* = 6. The variable *x*_*B*(*r*)_ = *x*_*k*_ = *x*_8_ is leaving the basis. We can now move on, to select the entering variable *x*_*l*_:

\begin{eqnarray*}{H}_{rP}& ={B}^{-1}{A}_{.P}=[2]\gt 0 \end{eqnarray*}

\begin{eqnarray*}{H}_{rQ}& ={B}^{-1}{A}_{.Q}=[-31]\lt 0 \end{eqnarray*}

\begin{eqnarray*}l& =2,t2=1,P=[6],Q=[8] \end{eqnarray*}

\begin{eqnarray*}{\theta }_{1}& =[~~],{\theta }_{2}=[0.0726] \end{eqnarray*}

Since only *θ*_2_ could be computed, the selection is done using the set *Q*. Variable *x*_*l*_ = *x*_2_ is entering the basis. The pivot operation now updates the basic and non-basic index lists as shown below:

\begin{eqnarray*}\overline{B}= \left[ \begin{array}{@{}c@{}} \displaystyle 3,4,5,7,1,2 \end{array} \right] ,\overline{N}= \left[ \begin{array}{@{}c@{}} \displaystyle 8,6 \end{array} \right] ,({A}_{B})^{-1}= \left[ \begin{array}{@{}cccccc@{}} \displaystyle 1&\displaystyle 0&\displaystyle 0&\displaystyle -0.0242&\displaystyle 0&\displaystyle -0.1371\\ \displaystyle 0&\displaystyle 1&\displaystyle 0&\displaystyle 0.0242&\displaystyle 0&\displaystyle 0.1371\\ \displaystyle 0&\displaystyle 0&\displaystyle 1&\displaystyle 0.4435&\displaystyle 0&\displaystyle -0.1532\\ \displaystyle 0&\displaystyle 0&\displaystyle 0&\displaystyle -0.4758&\displaystyle 1&\displaystyle -0.3629\\ \displaystyle 0&\displaystyle 0&\displaystyle 0&\displaystyle -0.0403&\displaystyle 0&\displaystyle 0.1048\\ \displaystyle 0&\displaystyle 0&\displaystyle 0&\displaystyle -0.0645&\displaystyle 0&\displaystyle -0.323 \end{array} \right] ,\nonumber\\\displaystyle ({s}_{N})^{T}= \left[ \begin{array}{@{}c@{}} \displaystyle 0.0726,-0.1048 \end{array} \right] ,{w}^{T}= \left[ \begin{array}{@{}c@{}} \displaystyle 0,0,0,0.1048,0,-0.0726\\ \displaystyle \end{array} \right] ,\nonumber\\\displaystyle {x}_{B}= \left[ \begin{array}{@{}c@{}} \displaystyle 2.1452\\ \displaystyle 3.8548\\ \displaystyle 19.3387\\ \displaystyle -2.1452\\ \displaystyle 1.2419\\ \displaystyle 1.3871 \end{array} \right] ,{\overline{x}}_{current}= \left[ \begin{array}{@{}c@{}} \displaystyle 1.2419\\ \displaystyle 1.3871\\ \displaystyle 2.1452\\ \displaystyle 3.8548\\ \displaystyle 19.3387\\ \displaystyle 0\\ \displaystyle -2.1452\\ \displaystyle 0 \end{array} \right] \end{eqnarray*}

Note that *x*_*B*_ ≤ 0 in this pivot. The new direction $\overline{d}={\overline{x}}_{middle}-{\overline{x}}_{current}$ now is:

\begin{eqnarray*}\overline{d}= \left[ \begin{array}{@{}c@{}} \displaystyle 0.8921\\ \displaystyle 2.7618\\ \displaystyle 3.8697\\ \displaystyle 2.1303\\ \displaystyle 13.5145\\ \displaystyle 16.4724\\ \displaystyle 9.2027\\ \displaystyle 9.6726 \end{array} \right] - \left[ \begin{array}{@{}c@{}} \displaystyle 1.2419\\ \displaystyle 1.3871\\ \displaystyle 2.1452\\ \displaystyle 3.8548\\ \displaystyle 19.3387\\ \displaystyle 0\\ \displaystyle -2.1452\\ \displaystyle 0 \end{array} \right] = \left[ \begin{array}{@{}c@{}} \displaystyle -0.3498\\ \displaystyle 1.3747\\ \displaystyle 1.7246\\ \displaystyle -1.7246\\ \displaystyle -5.8242\\ \displaystyle 16.4724\\ \displaystyle 11.3479\\ \displaystyle 9.6726 \end{array} \right] \Longrightarrow \overline{{d}_{B}}= \left[ \begin{array}{@{}c@{}} \displaystyle 1.7246\\ \displaystyle -1.7246\\ \displaystyle -5.8242\\ \displaystyle 11.3479\\ \displaystyle -0.3498\\ \displaystyle 1.3747\\ \displaystyle \end{array} \right] \end{eqnarray*}

**General loop: Iteration 2**

**Figure 4:**
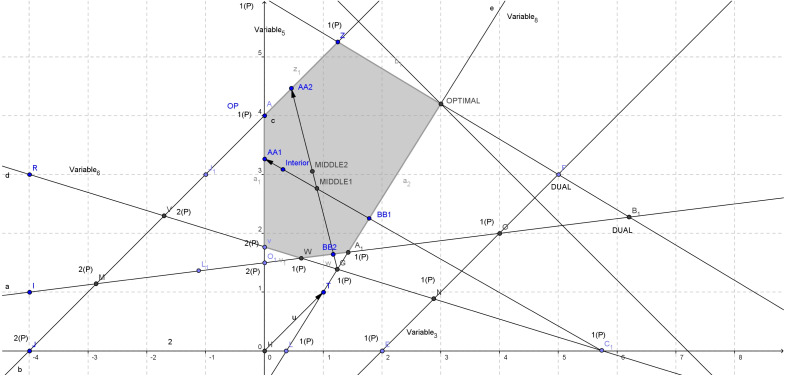
Constructing the second direction for Eq. (4).

The new exiting boundary point *AA*2 (as shown in Fig. 4) is:

\begin{eqnarray*}\alpha = \frac{{x}_{B[r]}}{-{d}_{B[r]}} =min \left\{ \frac{{x}_{B[i]}}{-{d}_{B[i]}} :{d}_{B[i]}\lt 0 \right\} & =& min \left\{ \frac{{x}_{B}[2,3,5]}{-{d}_{B}[2,3,5]} \right\} =min \left\{ \frac{3.8548}{1.7246} , \frac{19.3387}{5.8242} , \frac{1.2419}{0.3498} \right\} \nonumber\\\displaystyle & =& min\{2.2352,3.3204,3.5499\}=2.2352 \end{eqnarray*}

\begin{eqnarray*}\alpha \alpha ={x}_{current}+\alpha d= \left[ \begin{array}{@{}c@{}} \displaystyle 1.2419\\ \displaystyle 1.3871\\ \displaystyle 2.1452\\ \displaystyle 3.8548\\ \displaystyle 19.3387\\ \displaystyle 0\\ \displaystyle -2.1452\\ \displaystyle 0 \end{array} \right] +2.2352 \left[ \begin{array}{@{}c@{}} \displaystyle 0.3498\\ \displaystyle 1.3747\\ \displaystyle 1.7246\\ \displaystyle -1.7246\\ \displaystyle -5.8242\\ \displaystyle 16.4724\\ \displaystyle 11.3479\\ \displaystyle 9.6726 \end{array} \right] = \left[ \begin{array}{@{}c@{}} \displaystyle 0.4599\\ \displaystyle 4.4599\\ \displaystyle 6\\ \displaystyle 0\\ \displaystyle 6.3203\\ \displaystyle 36.8196\\ \displaystyle 23.2200\\ \displaystyle 21.6204 \end{array} \right] \end{eqnarray*}

Correspondingly, the entering point *BB*2 (as shown in Fig. 4) can be calculated using the maximum ratio test:

\begin{eqnarray*}\beta = \frac{-{x}_{B[r]}}{{d}_{B[r]}} =max \left\{ \frac{-{x}_{B[i]}}{{d}_{B[i]}} :{x}_{B[i]}\lt 0 \right\} =max \left\{ \frac{-{x}_{B}[4]}{{d}_{B}[4]} \right\} =max \left\{ \frac{-2.1452}{-11.3479} \right\} =0.1890 \end{eqnarray*}

Hence, *r*_*b*_ = 4  so  *r* = 4. The entering point then is:

\begin{eqnarray*}\beta \beta ={x}_{current}+\beta d= \left[ \begin{array}{@{}c@{}} \displaystyle 1.2419\\ \displaystyle 1.3871\\ \displaystyle 2.1452\\ \displaystyle 3.8548\\ \displaystyle 19.3387\\ \displaystyle 0\\ \displaystyle -2.1452\\ \displaystyle 0 \end{array} \right] +0.1890 \left[ \begin{array}{@{}c@{}} \displaystyle -0.3498\\ \displaystyle 1.3747\\ \displaystyle 1.7246\\ \displaystyle -1.7246\\ \displaystyle -5.8242\\ \displaystyle 16.4724\\ \displaystyle 11.3479\\ \displaystyle 9.6726 \end{array} \right] = \left[ \begin{array}{@{}c@{}} \displaystyle 1.1758\\ \displaystyle 1.6470\\ \displaystyle 2.4712\\ \displaystyle 3.5288\\ \displaystyle 18.2377\\ \displaystyle 3.1139\\ \displaystyle 0\\ \displaystyle 1.8285 \end{array} \right] \end{eqnarray*}

The new middle point *MIDDLE*2 (as shown in Fig. 4) between *αα* − *ββ* is now:

\begin{eqnarray*}{\overline{x}}_{middle}= \frac{\alpha \alpha +\beta \beta }{2} = \left[ \begin{array}{@{}c@{}} \displaystyle 0.8179\\ \displaystyle 3.0535\\ \displaystyle 4.2356\\ \displaystyle 1.7644\\ \displaystyle 12.2790\\ \displaystyle 19.9667\\ \displaystyle 11.6100\\ \displaystyle 11.7244 \end{array} \right] \end{eqnarray*}

The variable *x*_*B*(*r*)_ = *x*_*k*_ = *x*_7_, *r* = 4 is leaving the basis. We can now move on to select the entering variable *x*_*l*_:

\begin{eqnarray*}P& =[6],Q=[8] \end{eqnarray*}

\begin{eqnarray*}{H}_{rP}& =({A}_{B})^{-1}{A}_{.P}=[-0.4758]\lt 0 \end{eqnarray*}

\begin{eqnarray*}{H}_{rQ}& =({A}_{B})^{-1}{A}_{.Q}=[-0.3629]\lt 0 \end{eqnarray*}

\begin{eqnarray*}l& =2,t2=1,P=[7],Q=[8] \end{eqnarray*}

\begin{eqnarray*}{\theta }_{1}& =[-0.2203],{\theta }_{2}=[0.2] \end{eqnarray*}

Since *θ*_1_ ≤ *θ*_2_ the selection is done using the set *P*. Variable *x*_*l*_ = *x*_6_ is entering the basis. The pivot operation now updates the basic and non-basic index lists as shown below:

\begin{eqnarray*}\overline{B}& = \left[ \begin{array}{@{}c@{}} \displaystyle 3,4,5,6,1,2 \end{array} \right] ,\overline{N}= \left[ \begin{array}{@{}c@{}} \displaystyle 8,7 \end{array} \right] \end{eqnarray*}

\begin{eqnarray*}({A}_{B})^{-1}& = \left[ \begin{array}{@{}cccccc@{}} \displaystyle 1&\displaystyle 0&\displaystyle 0&\displaystyle 0&\displaystyle -0.0508&\displaystyle -0.1186\\ \displaystyle 0&\displaystyle 1&\displaystyle 0&\displaystyle 0&\displaystyle 0.0508&\displaystyle 0.1186\\ \displaystyle 0&\displaystyle 0&\displaystyle 1&\displaystyle 0&\displaystyle 0.9322&\displaystyle -0.4915\\ \displaystyle 0&\displaystyle 0&\displaystyle 0&\displaystyle 1&\displaystyle -2.1017&\displaystyle 0.7627\\ \displaystyle 0&\displaystyle 0&\displaystyle 0&\displaystyle 0&\displaystyle -0.0847&\displaystyle 0.1356\\ \displaystyle 0&\displaystyle 0&\displaystyle 0&\displaystyle 0&\displaystyle -0.1356&\displaystyle 0.0169 \end{array} \right] ,({s}_{N})^{T}= \left[ \begin{array}{@{}c@{}} \displaystyle 0.1525,-0.2203 \end{array} \right] , \end{eqnarray*}

\begin{eqnarray*}{w}^{T}& = \left[ \begin{array}{@{}c@{}} \displaystyle 0,0,0,0,0.2203,-0.1525 \end{array} \right] ,{x}_{B}= \left[ \begin{array}{@{}c@{}} \displaystyle 2.2542\\ \displaystyle 3.7458\\ \displaystyle 17.3390\\ \displaystyle 4.5085\\ \displaystyle 1.4237\\ \displaystyle 1.6780 \end{array} \right] \end{eqnarray*}

Note that *x*_*B*_ ≥ 0 in this pivot. Method iEPSA stops here. The basic solutions constructed as shown in Fig. 4 are *C*_1_ → *G* → *A*_1_. In practice, EPSA would take over and finish the optimization moving with one extra iteration to the optimal vertex from the feasible vertex *A*_1_. Note that in the second pivot, the middle point (*MIDDLE*2) constructed could have worse objective function than *MIDDLE*1 (although it seems better in this case). We did not calculate it on purpose here, since the second basic solution constructed is feasible. The sequence of the objective function value at each pair of basic solutions with the corresponding interior points is shown below:

\begin{eqnarray*}\text{basic solutions}:& \left[ \begin{array}{@{}ccc@{}} \displaystyle 5.75,&\displaystyle 2.629,&\displaystyle 3.1017 \end{array} \right] \end{eqnarray*}

\begin{eqnarray*}\text{interior points}:& \left[ \begin{array}{@{}c@{}} \displaystyle 3.4066\rightarrow improved\rightarrow 3.6539\\ \displaystyle 3.6539\rightarrow improved\rightarrow 3.8714 \end{array} \right] . \end{eqnarray*}

### Proof of Correctness

The proposed method consists of three different algorithms. Besides iEPSA (the non-monotonic part), the rest two (EPSA and PDIPSA) have been already proven to be correct and finite. For more details see (Paparrizos, Samaras & Stephanides, 2003a; Paparrizos, Samaras & Stephanides, 2003b). Since the nature of the iEPSA is non-monotonic, the finiteness cannot be verified by proving that the algorithm improves the objective function value at each iteration. Therefore iEPSA’s finiteness relies on the non-cycling property.

We will now use the same notation used in Zhang (1999) in respect to sign-properties and simplex tableau. Later on we will adjust to what is proven in Fukuda & Terlaky (1997) and explain how this portion of information affects iEPSA’s finiteness. With respect to the basic partition [*BN*] we call the following augmented matrix a simplex tableau:


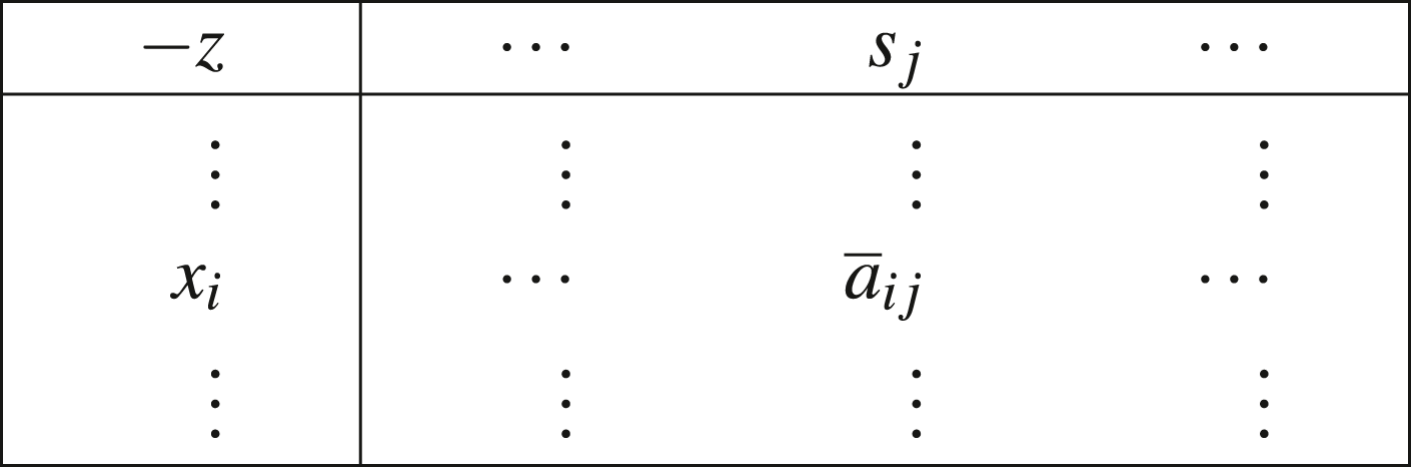


where:

\begin{eqnarray*}{ \left\vert {\overline{a}}_{ij} \right\vert }_{ \left\vert B \right\vert \times \left\vert N \right\vert }& =({A}_{B})^{-1}{A}_{N} \end{eqnarray*}

\begin{eqnarray*}\{{s}_{j}{|}j\in N\}& =({c}_{N})^{T}-({c}_{B})^{T}({A}_{B})^{-1}{A}_{N} \end{eqnarray*}

\begin{eqnarray*}\{{x}_{i}{|}i\in B\}& =({A}_{B})^{-1}b \end{eqnarray*}

\begin{eqnarray*}z& =({c}_{B})^{T}({A}_{B})^{-1}b \end{eqnarray*}

It is well known, that if *x*_*B*_ ≥ 0 the basis is primal feasible. If *s*_*N*_ ≥ 0 the basis is dual feasible. If both apply, the current basis is optimal. No primal or dual feasibility is required in iEPSA, and the value of the objective function does not improve necessarily in a monotonic way. Let us start by focusing on the correctness first. We have to prove that at each iteration, an available pivot is always given. This actually means that both the selection of the entering and leaving variables are well defined. First let us provide a series of proofs about the monotonicity of the interior point that iEPSA uses to construct the feasible direction at each iteration.

Lemma 1 A direction from an infeasible (exterior) point to an interior one, intersects the feasible region into a unique pair of entering/leaving points.

Proof Assume the polyhedron *X* = {*x*|*Ax* ≥ *b*}. Let points *αα*, *ββ* be computed as presented in iEPSA algorithm:

\begin{eqnarray*}\alpha \alpha & ={x}_{current}+\alpha d \end{eqnarray*}

\begin{eqnarray*}\beta \beta & ={x}_{current}+\beta d \end{eqnarray*}

Since *x*_*interior*_ and points *αα*, *ββ* lie on the same direction, the linear combination of the interior point is:

\begin{eqnarray*}{x}_{interior}=\lambda \alpha \alpha +(1-\lambda )\beta \beta \gt 0,~~\lambda \in (0,1). \end{eqnarray*}

So for each element *i* of *x*_*interior*_ the following applies:

\begin{eqnarray*}(\lambda a{a}_{i}+(1-\lambda )b{b}_{i})& \gt 0,(\lambda \gt 0),\forall i=1,...,m \end{eqnarray*}

\begin{eqnarray*}(a{a}_{i}+b{b}_{i})& \gt 0 \end{eqnarray*}

so there is no index *i* for which both elements in *αα*, *ββ* are equal to zero. This means that points *αα*, *ββ* lie on a different hyperplane of the feasible region.

Lemma 2 * Given a pair of a primal infeasible basic solution x*_*current*_* and an interior point x*_*interior*_*, the direction d* = *x*_*interior*_ − *x*_*current*_* intersects the feasible region into a pair of leaving/entering αα*, *ββ boundary points and the middle point* ${x}_{middle}= \frac{\alpha \alpha +\beta \beta }{2} $* is also interior.*

Proof We will use the contradiction method. Assume that *x*_*middle*_ is not interior. From Lemma1 we know that the entering *ββ* ≥ 0 and leaving *αα* ≥ 0 boundary points are not the same. For the middle point we have:

\begin{eqnarray*}{x}_{middle}= \frac{\alpha \alpha +\beta \beta }{2} \geq 0 \end{eqnarray*}

Since *x*_*middle*_ is not an interior point, there exists at least one element *i* equal to zero:

\begin{eqnarray*} \frac{\alpha {\alpha }_{i}+\beta {\beta }_{i}}{2} =0 \end{eqnarray*}

which contradicts with Lemma1. So *x*_*middle*_ is interior (*x*_*middle*_ > 0).

Lemma 3 From a given interior point, the half step of the minimum ratio test computed for any given descending direction results to a strictly better interior point.

Proof Let *x*_*interior*_ be the first interior point. For a given descending direction *d*, its widely know that the minimum ratio test computes the largest step we can move alongside the direction *d* while preserving feasibility. We get:

\begin{eqnarray*}\alpha = \frac{{x}_{B[{r}_{a}]}}{-{d}_{B[{r}_{a}]}} =min \left\{ \frac{{x}_{B[i]}}{-{d}_{B[i]}} :{d}_{B[i]}\lt 0 \right\} ,\forall i=1,2,...,m. \end{eqnarray*}

The new interior point is: ${\overline{x}}_{interior}={x}_{interior}+ \frac{\alpha }{2} d$. Suppose now that ${\overline{x}}_{interior}$ is not strictly better. We have:

\begin{eqnarray*}{c}^{T}{x}_{interior}& \leq {c}^{T}{\overline{x}}_{interior}\Leftrightarrow \end{eqnarray*}

\begin{eqnarray*}{c}^{T}{x}_{interior}-{c}^{T}{\overline{x}}_{interior}& \leq 0\Leftrightarrow \end{eqnarray*}

\begin{eqnarray*}{c}^{T}({x}_{interior}-{\overline{x}}_{interior})& \leq 0 \end{eqnarray*}

If we substitute ${\overline{x}}_{interior}$ we take:

\begin{eqnarray*}{c}^{T}({x}_{interior}-{x}_{interior}- \frac{\alpha }{2} d)& \leq 0\Leftrightarrow - \frac{\alpha }{2} {c}^{T}d\leq 0 \end{eqnarray*}

which is a contradiction since *d* < 0. Hence, the new interior point ${\overline{x}}_{interior}$ has a better objective value.

Lemma 4 At each iteration of iEPSA, the direction d intersects the feasible region.

Proof Lemmas 1, 2 and 3 immediately imply that at each iteration we construct the direction towards an interior point. Thus, each direction intersects the feasible region.

Theorem 1 * At each iteration of iEPSA, the objective value at the interior point x*_*interior*_* strictly decreases.*

Proof iEPSA starts by constructing the direction towards an interior point. Lemma 1 implies that the entering and leaving points for this direction are not the same. The algorithm then constructs at each iteration the middle point of the entering feasible ray segment. Lemma 2 shows that this point will also be interior. It then compares the two interior points and acts accordingly to secure the construction of a better interior point. By exploiting the non-monotonicity on the infeasible basic solutions that result in middle-interior points worse than in previous iterations and as a result offering a descending direction between these two we can still provide improvement on the interior point. Lemma 3 promotes the monotonicity in the interior of the feasible region.

We will prove each case by induction. The possible combinations for the objective function value between points *x*_*middle*_ and *x*_*interior*_ are shown below:

• *c*^*T*^*x*_*middle*_ < *c*^*T*^*x*_*interior*_: This case is trivial to examine. We directly acquire a better interior point due to the relational geometric position of the points.
• *c*^*T*^*x*_*middle*_ = *c*^*T*^*x*_*interior*_: Since the objective function’s value is same on both points, they either match or lie on a hyperplane vertical to the objective function vector *c*. We use *d* =  − *c* in that case, since the latter is a descending direction, as no direction can be constructed between the two points. Using Lemma 2 the new interior point will have better objective function value than the previous one.
• *c*^*T*^*x*_*middle*_ > *c*^*T*^*x*_*interior*_: We have
  \begin{eqnarray*}{c}^{T}{x}_{middle}& \gt {c}^{T}{x}_{interior}\Leftrightarrow \end{eqnarray*}
  \begin{eqnarray*}{c}^{T}{x}_{interior}-{c}^{T}{x}_{middle}& \lt 0\Leftrightarrow \end{eqnarray*}
  \begin{eqnarray*}{c}^{T}({x}_{interior}-{x}_{middle})& \lt 0\Leftrightarrow \end{eqnarray*}
  \begin{eqnarray*}{c}^{T}d& \lt 0 \end{eqnarray*}
  So it stands that the direction *d* = *x*_*interior*_ − *x*_*middle*_ is a descending direction. According to Lemma 3 the interior point which will be constructed in the next iteration using the half of the minimum ratio step from point *x*_*interior*_ will be better. So in this case a better interior point is also constructed.

For all the possible combinations an improved interior point can be constructed. Thus iEPSA uses monotonicity in interior points.

Lemma 5 At each iteration of iEPSA, a leaving variable is always eligible.

Proof Assume that at some iteration, *x*_*interior*_ > 0 is the current point and *x*_*current*_ < 0 is the current infeasible basic solution. From the constraints of Eq. (1) we have:

\begin{eqnarray*}(A{x}_{current}& =b and A{x}_{interior}=b)\Leftrightarrow \end{eqnarray*}

\begin{eqnarray*}A{x}_{interior}& =A{x}_{current}\Leftrightarrow \end{eqnarray*}

\begin{eqnarray*}A{x}_{interior}-A{x}_{current}& =0\Leftrightarrow \end{eqnarray*}

\begin{eqnarray*}A({x}_{interior}-{x}_{current})& =0\Leftrightarrow \end{eqnarray*}

\begin{eqnarray*}Ad& =0 \end{eqnarray*}

hence *d* is a direction. The maximum ratio test is then given by:

\begin{eqnarray*}\beta = \frac{-{x}_{B[r]}}{{d}_{B[r]}} =max \left\{ \frac{-{x}_{B[i]}}{{d}_{B[i]}} :{x}_{B[i]}\lt 0 \right\} ,\forall i=1,2,...m \end{eqnarray*}

and we now need to prove that *β* > 0. We have *d* = *x*_*interior*_ − *x*_*current*_, and for *x*_*current*_ < 0 since *x*_*interior*_ > 0, it follows that *d* > 0. Thus there exist columns in the maximum ratio test that will be positive, so *β* > 0 is computable.

Lemma 6 * If a pivot from an infeasible basic solution B* → *B*′* to another is admissible for iEPSA, then the inverse B*′ → *B is not.*

Proof First we will analyze the sign properties of the algorithm. Assume that *k* is the leaving variable (*k* ∈ *B*), *l* is the entering one (*l* ∈ *N*). The difference in objective function’s value between two consecutive extreme points $ \left[ B,N \right] $ and $ \left[ {B}^{{^{\prime}}},{N}^{{^{\prime}}} \right] $ is given by:

\begin{eqnarray*}{z}^{{^{\prime}}}-z=\Delta z=({s}_{N})_{l}({x}_{{B}^{{^{\prime}}}})_{l}. \end{eqnarray*}

We know also that the revised updating equation for the basic solution *x*_*B*_ is:

\begin{eqnarray*}{x}_{{B}^{{^{\prime}}}}& ={x}_{B}- \frac{f}{g} {h}_{l},\text{where} \end{eqnarray*}

\begin{eqnarray*}f& ={x}_{B(r)},g={H}_{rl},{h}_{.l}=({A}_{B})^{-1}{A}_{.l} \end{eqnarray*}

The algorithm selects the entering variable so as *H*_*rN*_ = [*H*_*rP*_*H*_*rQ*_] < 0, thus *H*_*rl*_ = *g* < 0. This means that the conjunction of the pivot row and column, the *pivot element*, is always negative for iEPSA. For the pivot row it also applies that *H*_*rl*_ =  − 1, *x*_*B*(*r*)_ = *x*_*k*_ = 0. So:

\begin{eqnarray*}{x}_{B(l)^{{^{\prime}}}}& ={x}_{l}= \frac{f}{g} = \frac{\lt 0}{\lt 0} \gt 0. \end{eqnarray*}

This means that the leaving variable will be replaced by a positive one after the pivot. Since the pivot from *B*′ → *B* is reverse to *B* → *B*′, the leaving variable of the first pivot cannot be selected immediately as leaving for the next pivot since it needs to be negative (as a reminder, the leaving variable is selected always by using the maximum ratio test, thus always negative).

Lemma 7 If iEPSA selects the entering variable from set P then the step is monotonic. If it is from set Q then the step is non-monotonic.

Proof When the selection is done from sets *P* and *Q* respectively, and since:

\begin{eqnarray*}P& = \left\{ j\in N:{s}_{j}\lt 0 \right\} and Q= \left\{ j\in N:{s}_{j}\geq 0 \right\} \end{eqnarray*}

we have (via the positivity of the leaving variable on the adjacent basic solution of Lemma 6):

\begin{eqnarray*}P& \Longrightarrow \Delta z={z}^{{^{\prime}}}-z={s}_{l}{x}_{l}=(-)(+)\lt 0 \end{eqnarray*}

\begin{eqnarray*}Q& \Longrightarrow \Delta z={z}^{{^{\prime}}}-z={s}_{l}{x}_{l}=(+)(+)\gt 0 \end{eqnarray*}

**Figure 5:**
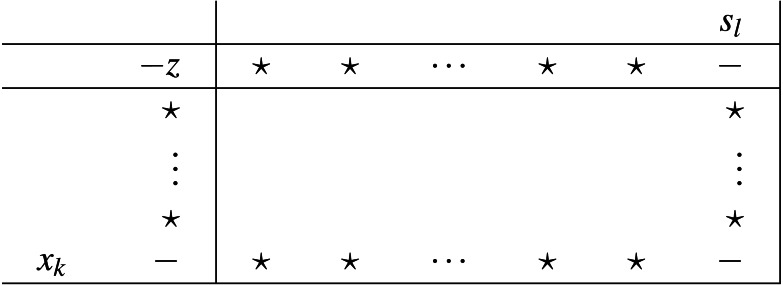
Pivot type I (set P).

**Figure 6:**
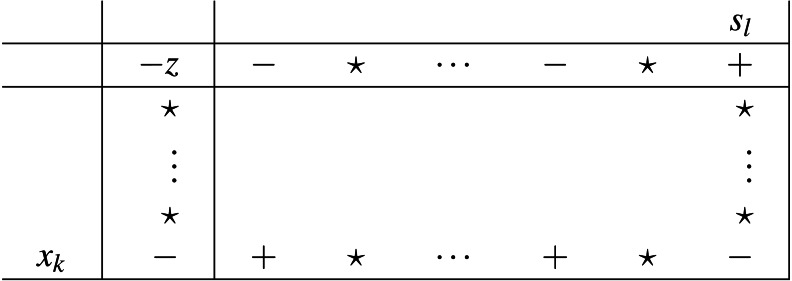
Pivot type II (set Q).

The sign properties previously proved result into the following unique pair of pivot types, depicted in Figs. 5 and 6. To select an entering variable from set *Q* (thus pivot of type II), automatically means that either *H*_*rP*_ ≥ 0, or *P* = Ø).

Lemma 8 The selection of the entering variable for iEPSA is well defined.

In Fukuda & Terlaky (1997) three types of terminal tableau for a linear problem are defined. Those are considered to be terminal because they define three different terminal states for a LP: (i) optimality (ii) primal-inconsistency (iii) dual-inconsistency. We emphasize on the second one shown here:


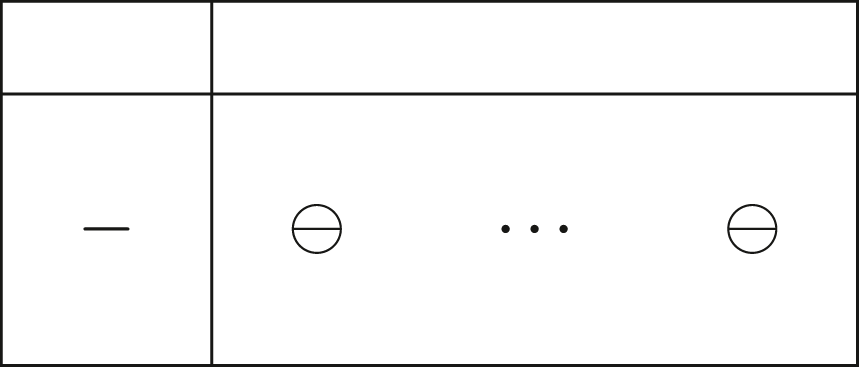

Proof Notice that since the leaving variable *x*_*k*_ = *x*_*B*(*r*)_ is always negative for iEPSA, and in Fukuda & Terlaky (1997) *D* =  − (*A*_*B*_)^−1^*A*_*N*_, this tableau version has opposite signs of what iEPSA uses for the pivot row (${H}_{rN}=({A}_{B})_{r}^{-1}{A}_{N}$). We know that iEPSA selects an entering variable with *H*_*rN*_ = [*H*_*rP*_*H*_*rQ*_] < 0, thus the pivot row on this terminal tableau matches the case where *H*_*rN*_ ≥ 0 for iEPSA. This would be a deadlock for iEPSA, a case where no eligible entering variable could be detected (all pivot elements positive or zero). As a result, this terminal tableau cannot occur in iEPSA run time, since iEPSA is only applicable on feasible LPs as it requires an initial interior point to initialize, and this terminal tableau reveals infeasibility of the primal problem.

Following the insight of what is stated in Fukuda & Terlaky (1997), we will now prove that iEPSA will never reach a deadlock, a case where the method is forced to cycle. We first need a definition:

Definition: *We call redundant a constraint in a LP which represents geometrically a half-space implied already by other constraint(s). Thus, a redundant constraint can be eliminated from the original LP without altering the equivalence of its initial feasible region.*

Theorem 2 Method iEPSA cannot reach a cycling deadlock.

Proof The near-terminal tableau of type B as shown in Fig. 7 in Fukuda & Terlaky (1997), actually means that the entering variable represents a redundant constraint. However in terms of iEPSA sign properties, this translates into: (i) negative leaving variable *x*_*k*_ (ii) pivot row: ${H}_{rN}=({A}_{B})_{r}^{-1}{A}_{N}\geq 0$ except for the entering variable *x*_*l*_, which gives (*H*_*rN*_)_.*l*_ < 0. This means that only one entering variable is eligible. We now move onto proving that vector *H*_*rN*_ cannot contain only one negative element in the general case of cycling. The cycling example that we will analyze is minimal; each variable in the cycle became entering and leaving only once.

This near-terminal tableau means that the constraint represented by variable *l* is a redundant constraint for the primal problem. Let us assume the general case, where cycling occurred as shown in Fig. 8. For simplicity, we depict basic solutions as nodes in a graph. Each oriented arrow represents an admissible pivot for iEPSA except for the one(s) in red color. A cycling assumption implies a case where the algorithm began on basic solution like node 1, moved after a finite number of pivots to node *n* and then again to 1, thus producing a cycle. All variables involved into the cycle changed basic/nonbasic status at least once.

Assume the pivot from (1 → *n*) is given by the pivot operation *x*_*k*_, *s*_*l*_ where *k* is the index of the leaving variable, and *l* the index of the entering one. The step (*n* → 1) was admissible by the algorithm, the sign properties apply, so on basic solution 1, *x*_*k*_ > 0 , and of course on *n*, *x*_*l*_ < 0 (via Lemma 6). It is now also clear that (1 → *n*) cannot be admissible for the algorithm (although 1, *n* are obviously neighbors) via Lemma 6. However, somewhere before visiting node *n*, variable *k*, changed status and left the basis (in order to be eligible as entering on the last node *n*).

Now, since the algorithm always selects leaving variables throughout a max-ratio test, it’s obvious that all leaving variables are hyperplanes of the feasible region. Thus all leaving variables selected by the algorithm are *non-redundant*, as removing either of them would result to a new LP which would not be equivalent to the previous one.

If we assume that there is only one pivot admissible by the sign properties of the algorithm on node *n* (that is, moving to 1), this means that the tableau on that pivot has a negative leaving variable and, there is only one negative element in the pivot row *H*_*rN*_. However according to Fukuda & Terlaky (1997), this is a near-terminal tableau of type *B*. It means that the constraint that the entering variable represents, is a *redundant* one for the primal problem. Since the entering variable *k* on *n*, is already known to be leaving in some previous iteration, and all leaving variables are non-redundant in this algorithm this is a contradiction. This near-terminal tableau of type *B* tableau can never appear for an entering variable that has already previously served as an outgoing.

The algorithm as shown in Fig. 8 from *n* can either move to *n* → 1 or to 2, since *n* is a neighbor to 1 and 1 is a neighbor to 2, then *n* is potentially a neighbor to 2. If not a neighbor, the pivot would not be admissible anyhow to assume a case of cycling. Additionally, node *n* − 1 is excluded as via Lemma 6 the backwards pivot is non-admissible, since the forward was. The direct backwards pivot to *n* → 1 is not possible via Lemma 6. So the algorithm can again theoretically cycle with 2 now. We extract the following scenario depicted in Fig. 9: the pivots 1 → 2, *n* → 2, *n* → 1 in the case of cycling are all admissible. Via Lemma 6, we know that the leaving variable is always negative, and always being substituted by a positive one on the pivot. Since 1 → 2 is admissible, *k*_1_ ≤ 0. Since *n* → 2 is also admissible, *k*_2_ ≤ 0 as well. But in the pivot *n* → 1, variable *k* must be substituted at node 1 with a positive variable, and the substitution here is *k*_1_. However since 1 → 2 is admissible, again *k*_1_ ≤ 0 must apply; contradiction.

**Figure 7:**
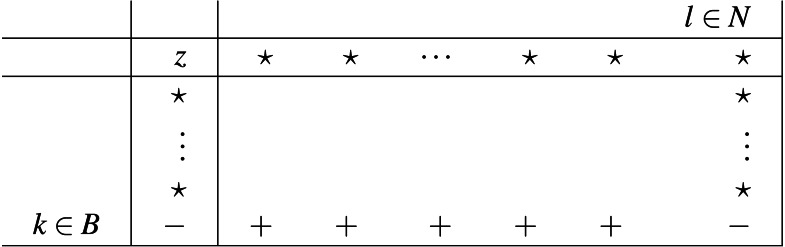
Near terminal tableau type B.

**Figure 8:**
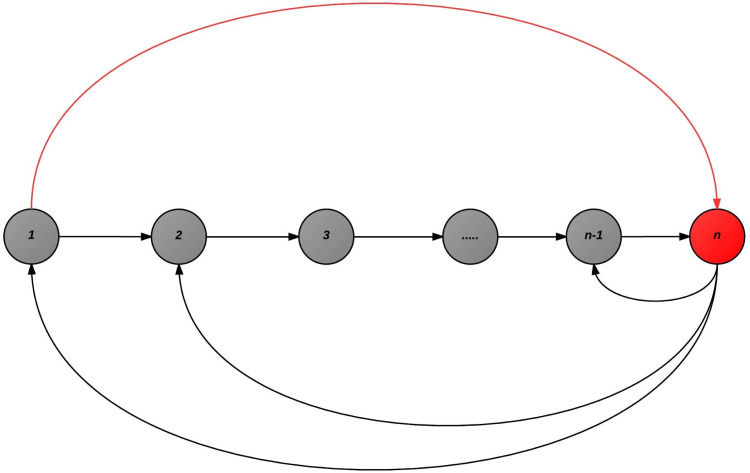
The general case of cycling.

**Figure 9:**
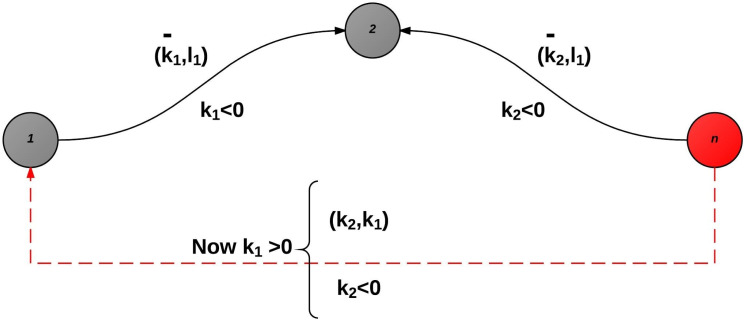
Supposing cycling was possible even for the only second alternative entering variable, this scheme must apply.

This case can only take place if 1 → 2, *n* → 2 are admissible pivots, but the pivot element for *n* → 1 is positive, which is not applicable for iEPSA, so as the returning variable *k*_1_ can be again negative. This means that the pivot *n* → 1 is both ways non-admissible for iEPSA if this applies.

## Results

Computational studies can provide preliminary results on the computational behavior of an algorithm, thus enabling us to gain insight of its performance in different types of LPs. The most popular types of test instances available for computational experiments are instances from real world applications. The test bed used in the computational study was a set of benchmark problems (netlib, Kennington, Mészáros) that do not have bounds and ranges sections in their *.mps* files. All reported times were measured in seconds with built-in function *cputime*.

The computing environment we used in the computational study is described in Table 1, using the most up-to-date possible software versions. Table 2 presents detailed information about the computational study. The first column includes the name of the problem, the second the number of constraints, the third the number of variables, the fourth the nonzero elements of the matrix *A* and then the triplets of the results for all three algorithms in terms of cpu time and total number of iterations follow. Finally the last three columns contain the optimal objective value reported by each algorithm. The test bed includes 93 LPs from netlib, Kennington and Mészáros collection. Ordónez & Freund (2003) have shown that nearly 71% of the netlib LPs are ill-conditioned. Hence, numerical difficulties may occur. We implemented an *mps* parser to read mps-files and convert data into MATLAB *mat* files.

**Figure 10:**
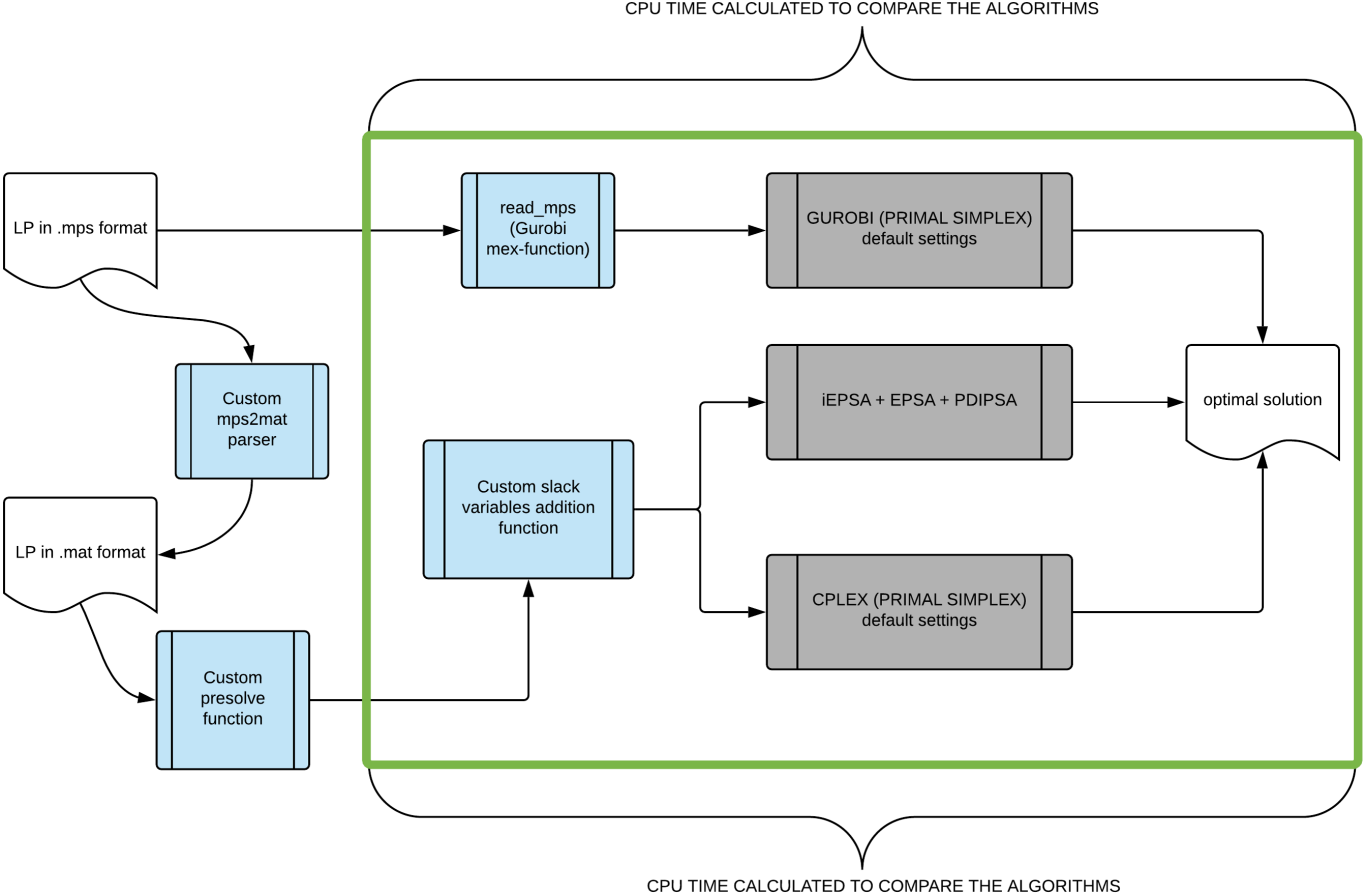
The pipeline showing how we gradually construct an appropriate .mat file format (MATLAB data file) to input in the competing algorithms and which segment of the pipeline we took into account in calculating their total cpu running time.

The proposed algorithm (iEPSA) was implemented in MathWorks MATLAB environment. The main reason for this choice was the support for high-level sparse matrix operations. Four programming adjustments were used to improve the performance of memory bound code in MATLAB. Those were: (i) store and access data in columns, (ii) avoid creating unnecessary variables, (iii) vectorization instead of for-loops and (iv) pre-allocate arrays before accessing them within loops. In order to handle numerical errors, tolerance scalars were introduced. The default values of the above mentioned tolerances were set equal to 1*e* − 8 for all vectors and matrices for iEPSA. For the basis update of iEPSA, EPSA and PDIPSA we used the MATLAB *LU* factorization decomposition scheme. Furthermore, to obtain the first (and only) interior point for iEPSA we used a MATLAB interface under a MEX-function provided for the MOSEK IPM (Andersen & Andersen, 2000; Andersen & Ye, 1996). This interface allowed us to modify appropriately the IPM so as to stop the solver directly after finding the first interior point. We emphasize that we used a zero objective function vector with MOSEK, and as a result MOSEK IPM is *agnostic* in directions pointing towards any existing optimal solutions. In this way, the claim that the first interior point we construct actually brings iEPSA very close to the optimal solution already, is at least unsubstantiated. The pipeline representing how the total running times were calculated for the competing algorithms is shown in Fig. 10. We did not use any scaling technique to solve successfully all tested benchmarks as the EPSA pivoting scheme is scaling invariant (Triantafyllidis & Samaras, 2014).

**Table 1:** Description of the computing environment.

CPU	Intel® XEON^™^ E-2186 (2.9Ghz @ 6 cores - 12 threads)
RAM Size	64GB 2666MHz DDR4 Memory
L3 cache size	12MB
Operating System	Windows 10 Pro x64
MATLAB version	R2019b (9.7.0.1216025) Update 1 (Build date: Sept. 2019)
MOSEK Interior-Point Method	v.9.0.94 (Build date: June 2019)
CPLEX Primal Simplex (ILOG Opt.Studio)	v.12.9 (Build date: March 2019)
Gurobi Primal Simplex	v.8.1.1

In Table 2 we present the arithmetic mean (*A*_*MEAN*) computed for both the total cpu time (in seconds) and number of iterations (niter). We present the execution time and the number of iterations of each algorithm over the netlib, Kennington and Mészáros set of LPs included. We compare the performance of the proposed new algorithm (iEPSA) against CPLEX (Primal Simplex) using its default settings and forcing the suite to use Primal Simplex (as iEPSA also solves the primal problem) and Gurobi (Primal Simplex) using the same method as well. The proposed algorithm performs fewer iterations on 44/93 benchmarks versus CPLEX and 43/93 versus Gurobi. Specifically, in terms of average number of iterations, iEPSA performs 47.3% less iterations than CPLEX and 28.7% less iterations than Gurobi.

**Table 2:** Computational results on a selection of well known benchmark LPs.

					**CPU (s)**			**NITER**			**OBJECTIVE VALUE**		
	**BENCHMARK**	**ROWS**	**COLUMNS**	**SPARSITY**	**CPLEX**	**iEPSA**	**GUROBI**	**CPLEX**	**iEPSA**	**GUROBI**	**CPLEX**	**iEPSA**	**GUROBI**
1	adlittle	55	95	7.18%	0.351	0.143	0.008	80	96	99	2.25E+05	2.25E+05	2.25E+05
2	afiro	26	32	9.74%	0.087	0.065	0.008	6	15	7	−4.65E+02	−4.65E+02	−4.65E+02
3	agg	112	112	4.94%	0.087	0.164	0.03	59	99	77	−1.64E+08	−1.64E+08	−1.64E+08
4	agg2	301	301	3.07%	0.084	0.284	0.019	117	201	170	−5.81E+07	−5.81E+07	−5.81E+07
5	agg3	301	301	3.08%	0.193	0.205	0.014	116	212	193	−3.83E+07	−3.83E+07	−3.83E+07
6	bandm	243	398	1.99%	0.084	0.351	0.012	266	307	308	−3.08E+02	−3.08E+02	−3.08E+02
7	baxter	14959	14959	0.03%	5.297	1.449	0.066	12	23	21	1.77E+15	1.77E+15	1.77E+15
8	beaconfd	82	143	10.70%	0.068	0.066	0.01	1	32	19	3.35E+04	3.35E+04	3.35E+04
9	blend	71	80	7.85%	0.073	0.089	0	77	106	50	−3.08E+01	−3.08E+01	−3.08E+01
10	bnl1	596	1,169	0.72%	0.136	1.359	0.027	1,897	874	1,446	1.88E+03	1.88E+03	1.88E+03
11	bnl2	1,821	3,007	0.23%	0.293	5.769	0.088	3,826	1,840	2,719	1.74E+03	1.74E+03	1.74E+03
12	brandy	133	207	6.89%	0.063	0.179	0.01	156	166	162	1.52E+03	1.52E+03	1.52E+03
13	cep1	1,520	3,248	0.14%	0.149	1.421	0.094	2,181	1,154	4,063	5.00E+04	5.00E+04	5.00E+04
14	cr42	905	1,513	0.48%	0.07	1.141	0.04	521	529	219	2.80E+01	2.80E+01	2.80E+01
15	cre_a	2,977	3,969	0.12%	0.443	4.989	0.041	2,904	1,973	2,962	2.36E+07	2.36E+07	2.36E+07
16	cre_c	2,349	3,392	0.14%	0.292	2.87	0.043	1,432	1,290	1,498	2.43E+07	2.43E+07	2.43E+07
17	degen2	442	534	1.67%	0.104	0.632	0.016	1,080	390	740	−1.44E+03	−1.44E+03	−1.44E+03
18	degen3	1,501	1,818	0.90%	0.415	7.486	0.051	4,549	1,560	4,276	−9.87E+02	−9.87E+02	−9.87E+02
19	e18	14230	14230	0.05%	4.877	11.58	0.12	655	727	1,282	3.00E+02	3.00E+02	3.00E+02
20	fffff800	319	663	2.36%	0.071	0.307	0.016	364	298	154	5.56E+05	5.56E+05	5.56E+05
21	fxm2-6	1,388	2,056	0.37%	0.127	2.749	0.061	1,433	1,415	1,615	1.84E+04	1.84E+04	1.84E+04
22	iiasa	632	2,970	0.35%	0.086	0.645	0.052	1,468	647	1,553	1.90E+08	1.90E+08	1.90E+08
23	israel	142	142	10.50%	0.071	0.247	0.007	171	295	145	−9.00E+05	−9.00E+05	−9.00E+05
24	large002	3,800	5,484	0.08%	0.684	32.964	0.177	4,749	1,941	2,167	7.61E+13	7.61E+13	8.61E−01
25	large003	3,726	5,457	0.08%	0.543	26.376	0.138	4,009	1,876	2,363	5.45E+17	5.45E+17	1.18E+02
26	large007	3,780	5,477	0.08%	0.64	30.559	0.184	4,031	2,456	2,475	6.30E+16	6.30E+16	1.62E+00
27	large008	3,798	5,484	0.08%	0.675	33.135	0.145	4,002	2,895	2,231	1.79E+16	1.79E+16	2.11E+00
28	large009	3,787	5,484	0.08%	0.667	33.223	0.143	4,236	3,048	2,337	1.76E+16	1.76E+16	1.77E+00
29	large011	3,782	5,480	0.08%	0.751	31.291	0.168	4,723	2,596	2,362	1.86E+16	1.86E+16	1.79E+00
30	large012	3,802	5,484	0.08%	0.669	31.915	0.146	4,270	2,359	2,160	4.13E+16	4.13E+16	1.78E+00
31	large016	3,810	5,458	0.08%	0.573	33.549	0.186	3,138	2,824	2,349	3.72E+21	3.72E+21	1.17E+02
32	lotfi	133	288	2.11%	0.068	0.14	0.006	97	204	162	−2.53E+01	−2.53E+01	−2.53E+01
33	multi	60	102	15.70%	0.055	0.032	0.036	53	49	64	4.04E+04	4.04E+04	4.04E+04
34	nemscem	479	1,398	0.50%	0.064	0.193	0.042	320	163	196	7.56E+04	7.56E+04	7.56E+04
35	nsic1	441	463	1.39%	0.062	0.727	0.037	382	418	398	−1.21E+07	−1.21E+07	−1.21E+07
36	nug05	148	225	2.22%	0.062	0.061	0.038	696	40	112	5.00E+01	5.00E+01	5.00E+01
37	nw14	73	123409	10.04%	0.394	10.392	0.403	179	302	4,895	6.18E+04	6.18E+04	6.18E+04
38	osa-07	1,118	23949	0.54%	0.428	2.136	0.11	681	445	1,112	5.36E+05	5.36E+05	5.36E+05
39	p0033	15	32	20.21%	0.062	0.011	0.025	16	13	14	1.73E+03	1.73E+03	1.73E+03
40	p0040	23	40	11.96%	0.062	0.015	0.025	6	14	16	6.18E+04	6.18E+04	6.18E+04
41	p0201	133	201	7.19%	0.064	0.053	0.009	64	72	114	6.88E+03	6.88E+03	6.88E+03
42	p0282	233	274	2.70%	0.07	0.014	0.006	33	10	105	3.67E+05	3.67E+05	3.67E+05
43	p0548	170	543	1.70%	0.069	0.089	0.008	88	101	71	3.77E+04	3.77E+04	3.77E+04
44	p19	284	586	3.19%	0.075	0.224	0.009	333	243	413	2.38E+05	2.38E+05	2.38E+05
45	p2756	739	2,740	0.39%	0.099	0.05	0.013	61	29	203	2.10E+04	2.10E+04	2.10E+04
46	refine	27	31	14.10%	0.065	0.022	0.005	24	24	17	−4.86E+05	−4.86E+05	−4.86E+05
47	rlfddd	4,050	57471	0.11%	2.811	0.115	0.048	13	14	13	−1.30E+01	−1.30E+01	−1.30E+01
48	rlfprim	8,048	8,048	0.04%	1.641	1.029	0.012	59	89	88	1.00E+00	1.00E+00	1.00E+00
49	route	20779	23923	0.04%	13.334	117.45	1.27	37650	7,551	20749	5.94E+03	5.94E+03	5.94E+03
50	sc105	103	103	2.62%	0.062	0.05	0	18	55	23	−5.43E+01	−5.43E+01	−5.43E+01
51	sc205	202	202	1.34%	0.061	0.147	0	76	200	63	−5.27E+01	−5.27E+01	−5.27E+01
52	sc50a	47	47	5.70%	0.059	0.018	0	7	25	8	−7.59E+01	−7.59E+01	−7.59E+01
53	sc50b	48	48	5.12%	0.058	0.03	0	11	44	12	−7.00E+01	−7.00E+01	−7.00E+01
54	scagr25	469	498	0.66%	0.068	0.267	0.014	350	181	517	−1.45E+07	−1.45E+07	−1.45E+07
55	scagr7	127	138	2.34%	0.061	0.039	0.005	84	43	71	−2.08E+06	−2.08E+06	−2.08E+06
56	scagr7-2r-108	3,474	4,123	0.08%	0.301	15.115	0.019	1,188	1,321	1,469	−8.34E+05	−8.34E+05	−8.34E+05
57	scagr7-2r-16	546	643	0.53%	0.065	0.262	0.008	130	191	228	−8.33E+05	−8.33E+05	−8.33E+05
58	scagr7-2r-27	909	1,072	0.32%	0.074	0.625	0.011	273	342	376	−8.34E+05	−8.34E+05	−8.34E+05
59	scagr7-2r-4	150	175	1.89%	0.056	0.048	0.007	68	54	90	−8.33E+05	−8.33E+05	−8.33E+05
60	scagr7-2r-8	282	331	1.02%	0.059	0.092	0.006	68	103	152	−8.33E+05	−8.33E+05	−8.33E+05
61	scfxm1	302	431	1.76%	0.065	0.244	0.01	283	232	288	1.85E+04	1.85E+04	1.85E+04
62	scfxm1-2b-4	622	946	0.59%	0.077	0.744	0.015	571	524	508	2.88E+03	2.88E+03	2.88E+03
63	scfxm1-2c-4	622	946	0.59%	0.083	0.765	0.015	595	538	558	2.88E+03	2.88E+03	2.88E+03
64	scfxm1-2r-4	622	946	0.59%	0.072	0.764	0.031	551	528	552	2.88E+03	2.88E+03	2.88E+03
65	scfxm1-2r-8	1,158	1,786	0.30%	0.103	2.348	0.045	1,127	1,115	1,252	2.88E+03	2.88E+03	2.88E+03
66	scfxm2	604	862	0.88%	0.077	0.77	0.02	582	553	573	3.68E+04	3.68E+04	3.68E+04
67	scfxm3	906	1,293	0.59%	0.098	1.411	0.023	875	801	947	5.50E+04	5.50E+04	5.50E+04
68	scorpion	317	324	1.13%	0.063	0.119	0.008	67	88	56	1.88E+03	1.88E+03	1.88E+03
69	scrs8	422	1,109	0.63%	0.073	0.403	0.009	515	375	218	9.04E+02	9.04E+02	9.04E+02
70	scrs8-2r-64b	936	1,587	0.20%	0.083	0.346	0.027	28	162	35	1.35E+03	1.35E+03	1.35E+03
71	scsd1	77	760	4.08%	0.065	0.271	0.004	258	358	216	8.67E+00	8.67E+00	8.67E+00
72	scsd8	397	2,750	0.79%	0.092	3.162	0.01	1,720	1,869	786	9.05E+02	9.05E+02	9.05E+02
73	scsd8-2b-4	90	630	3.33%	0.058	0.183	0.025	193	239	97	1.53E+01	1.53E+01	1.53E+01
74	scsd8-2c-16	330	2,310	0.94%	0.075	1.159	0.028	1,174	827	251	1.50E+01	1.50E+01	1.50E+01
75	scsd8-2c-4	90	630	3.33%	0.062	0.167	0.04	215	227	97	1.50E+01	1.50E+01	1.50E+01
76	scsd8-2r-4	90	630	3.33%	0.063	0.171	0.028	155	224	83	1.55E+01	1.55E+01	1.55E+01
77	scsd8-2r-8	170	1,190	1.80%	0.062	0.476	0.027	337	493	134	1.60E+01	1.60E+01	1.60E+01
78	scsd8-2r-8b	170	1,190	1.80%	0.063	0.52	0.028	337	493	134	1.60E+01	1.60E+01	1.60E+01
79	sctap1	284	480	1.20%	0.078	0.249	0.008	185	313	171	1.41E+03	1.41E+03	1.41E+03
80	sctap1-2b-16	990	1,584	0.37%	0.087	0.439	0.024	294	414	113	2.81E+02	2.81E+02	2.81E+02
81	sctap1-2b-4	270	432	1.30%	0.061	0.084	0.028	79	106	40	2.39E+02	2.39E+02	2.39E+02
82	sctap1-2c-16	990	1,584	0.37%	0.088	0.518	0.026	322	451	167	3.26E+02	3.26E+02	3.26E+02
83	sctap1-2c-4	270	432	1.30%	0.065	0.082	0.04	93	112	48	2.36E+02	2.36E+02	2.36E+02
84	sctap1-2r-4	270	432	1.30%	0.058	0.079	0.035	64	106	15	2.81E+02	2.81E+02	2.81E+02
85	sctap1-2r-8	510	816	0.70%	0.068	0.189	0.026	125	229	41	3.61E+02	3.61E+02	3.61E+02
86	sctap1-2r-8b	510	816	0.70%	0.067	0.169	0.026	134	198	43	2.50E+02	2.50E+02	2.50E+02
87	sctap2	1,033	1,880	0.33%	0.098	0.473	0.015	535	351	237	1.72E+03	1.72E+03	1.72E+03
88	sctap3	1,408	2,480	0.25%	0.123	0.794	0.018	721	465	315	1.42E+03	1.42E+03	1.42E+03
89	share1b	112	220	4.55%	0.061	0.088	0.009	141	98	207	−7.66E+04	−7.66E+04	−7.66E+04
90	share2b	79	79	9.85%	0.059	0.035	0.005	42	39	49	−6.60E+02	−6.60E+02	−6.60E+02
91	ship08s	276	1,582	0.83%	0.068	0.493	0.009	282	410	308	1.88E+06	1.88E+06	1.88E+06
92	slptsk	2,861	3,347	0.76%	0.498	55.496	0.366	3,216	1,210	2,789	2.99E+01	2.99E+01	2.99E+01
93	stocfor1	96	96	3.53%	0.064	0.044	0.005	11	51	21	−7.91E+04	−7.91E+04	−7.91E+04
**A_MEAN**					**0.452**	**5.59**	**0.057**	**1,240.688**	**653.581**	**917.441**			

**Figure 11:**
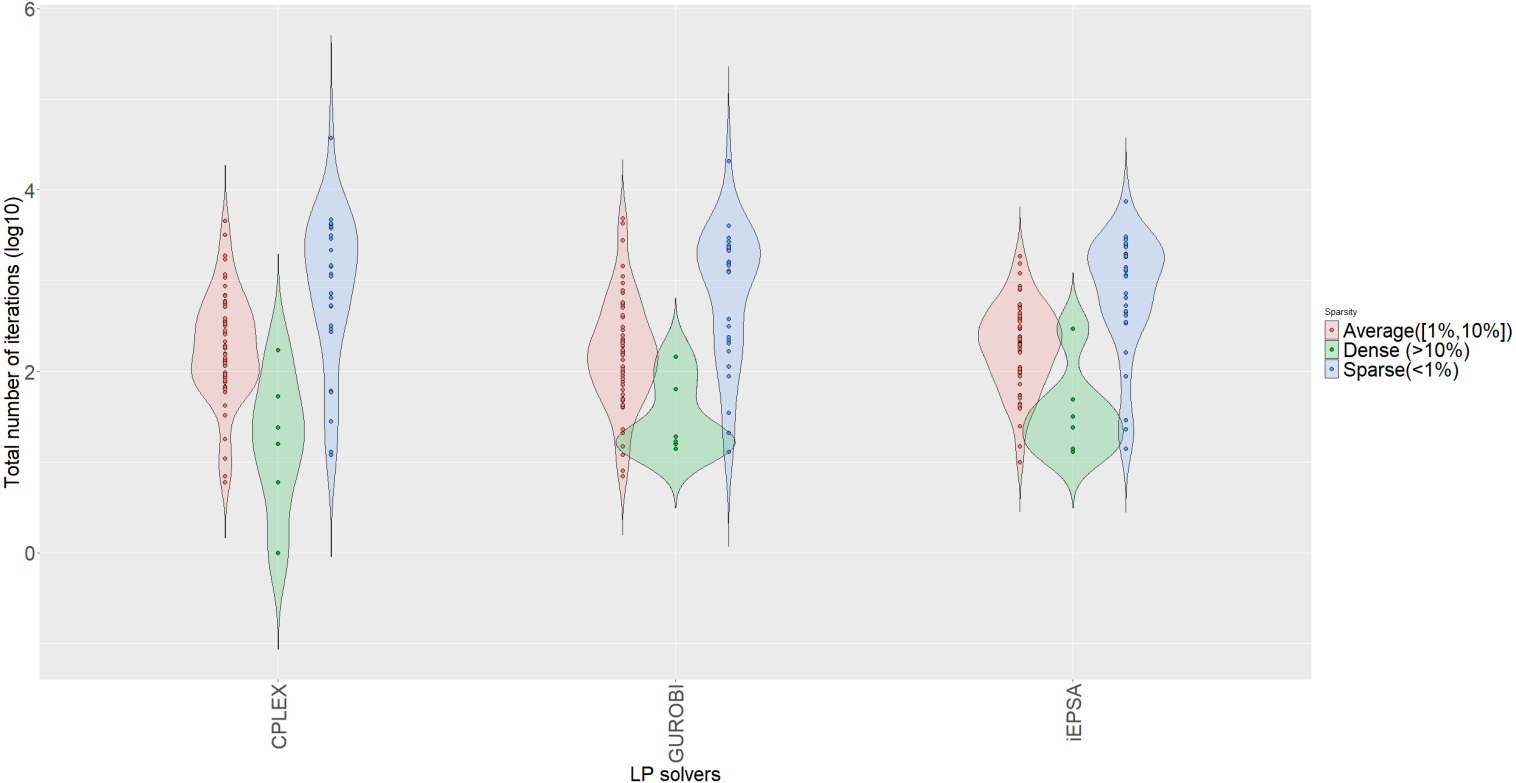
Violin plots of number of iterations stratified by sparsity for all algorithms.

In terms of cpu-time, CPLEX, iEPSA and Gurobi correspondingly required on average 0.452, 5.59 and 0.057 seconds to solve all tested instances. Even if the difference between iEPSA and the commercial solvers is substantial, it is worthy to note that m-code (iEPSA) is significantly slower than pure *C* implementations (mex-functions of CPLEX and Gurobi), thus justifiable to witness in this computational study. However, the average number of iterations is irrelevant to the programming language used, and only relies on the algorithmic mechanics for each solver. Therefore, it can be a transparent criterion to gain insight about practical performance among the competing algorithms. Also, Fig. 11 shows the violin plots for the total number of iterations for all three algorithms across all tested benchmarks stratified by three levels of sparsity.

Finally, there have been observed differences in the optimal value between the solvers in the problems of the family *largeXXX*. This has to do with the very nature of the problems themselves where numerical instability is highly present. iEPSA tends to agree completely though with CPLEX objective values.

## Conclusions

In this paper we proposed a new non-monotonic simplex-type algorithm for solving LPs. iEPSA does not maintain monotonicity on the basic solutions but only on the interior point solutions. This new algorithm is a combination of three different methods. The computational results are very encouraging for the new algorithm. Algorithm iEPSA performs 47.3% less iterations than CPLEX and 28.7% less iterations than Gurobi in a collection of 93 well-known benchmarks. Future work includes the extension of the computational studies to a larger number of tested problems from available benchmark collections, improving the cpu-time performance by implementing the algorithms to a low-level programming language and finally, implementing a tailored method to compute the first interior point rather than using a commercial IPM.

## Additional Information and Declarations

### Data Availability

The following information was supplied regarding data availability: Data is available at GitHub: https://github.com/harrytr/iEPSA_2019.

## References

[ref-1] Amin GR, Emrouznejad A (2011). Optimizing search engines results using linear programming. Expert Systems with Applications.

[ref-2] Andersen ED, Andersen KD, Frenk H, Roos K, Terlaky T, Zhang S (2000). The MOSEK interior point optimizer for linear programming: an implementation of the homogeneous algorithm. High performance optimization.

[ref-3] Andersen ED, Ye Y (1996). Combining interior-point and pivoting algorithms for linear programming. Management Science.

[ref-4] Arsham H (2007). A hybrid gradient and feasible direction pivotal solution algorithm for general linear programs. Applied Mathematics and Computation.

[ref-5] Basu A, Loera JAD, Junod M (2014). On Chubanov’s method for linear programming. INFORMS Journal on Computing.

[ref-6] Bertsimas D, Tsitsiklis JN (1997). Introduction to linear optimization.

[ref-7] Bixby RE, Gregory JW, Lustig IJ, Marsten RE, Shanno DF (1992). Very large-scale linear programming: a case study in combining interior point and simplex methods. Operations Research.

[ref-8] Bixby RE, Saltzman MJ (1994). Recovering an optimal LP basis from an interior point solution. Operations Research Letters.

[ref-9] Burdett RL, Kozan E, Sinnott M, Cook D, Tian YC (2017). A mixed integer linear programing approach to perform hospital capacity assessments. Expert Systems with Applications.

[ref-10] Dantzig GB (1949). Programming of interdependent activities: II mathematical model. Econometrica.

[ref-11] Dongarra J, Sullivan F (2000). Guest editors introduction: the top 10 algorithms. Computing in Science & Engineering.

[ref-12] Elhallaoui I, Metrane A, Desaulniers G, Soumis F (2011). An improved primal simplex algorithm for degenerate linear programs. INFORMS Journal on Computing.

[ref-13] Fernndez S, Borrajo D (2012). Using linear programming to solve clustered oversubscription planning problems for designing e-courses. Expert Systems with Applications.

[ref-14] Fukuda K, Terlaky T (1997). Criss-cross methods: a fresh view on pivot algorithms. Mathematical Programming.

[ref-15] Gkioulekas I, Papageorgiou LG (2019). Piecewise regression analysis through information criteria using mathematical programming. Expert Systems with Applications.

[ref-16] Glavelis T, Ploskas N, Samaras N (2018). Improving a primal-dual simplex-type algorithm using interior point methods. Optimization.

[ref-17] Gleixner AM, Steffy DE, Wolter K (2016). Iterative refinement for linear programming. INFORMS Journal on Computing.

[ref-18] Illes T, Terlaky T (2002). Pivot versus interior point methods: pros and cons. European Journal of Operational Research.

[ref-19] Janssens GK, Ramaekers KM (2011). A linear programming formulation for an inventory management decision problem with a service constraint. Expert Systems with Applications.

[ref-20] Jurik T (2008). A nearest point approach algorithm for a class of linear programming problems. Journal of Applied Mathematics, Statistics and Informatics (JAMSI).

[ref-21] Karmarkar N (1984). A new polynomial-time algorithm for linear programming.

[ref-22] Khachiyan LG (1979). A polynomial algorithm in linear programming. Doklady Akademii Nauk SSSR.

[ref-23] Klee V, Minty GJ, Shisha O (1972). How good is the simplex algorithm?. Inequalities.

[ref-24] Li W (2013). Dual–primal algorithm for linear optimization. Optimization Methods Software.

[ref-25] Mehrotra S (1992). On the implementation of a primal-dual interior point method. SIAM Journal on Optimization.

[ref-26] Mehrotra S (1993). Quadratic convergence in a primal-dual method. Mathematics of Operations Research.

[ref-27] Murty K, Fathi Y (1984). A feasible direction method for linear programming. Operations Research Letters.

[ref-28] Omer J, Rosat S, Raymond V, Soumis F (2015). Improved primal simplex: a more general theoretical framework and an extended experimental analysis. INFORMS Journal on Computing.

[ref-29] Ordónez F, Freund RM (2003). Computational experience and the explanatory value of condition measures for linear optimization. SIAM Journal on Optimization.

[ref-30] Pan PQ (2008). A largest-distance pivot rule for the simplex algorithm. European Journal of Operational Research.

[ref-31] Pan PQ (2013). An affine-scaling pivot algorithm for linear programming. Optimization.

[ref-32] Paparrizos K (1991). An infeasible (exterior point) simplex algorithm for assignment problems. Math Program.

[ref-33] Paparrizos K (1993). An exterior point simplex algorithm for (general) linear programming problems. Annals of Operations Research.

[ref-34] Paparrizos K (1996). A new primal and dual pivoting rule for the simplex algorithm.

[ref-35] Paparrizos K, Samaras N, Sifaleras A (2015). Exterior point simplex-type algorithms for linear and network optimization problems. Annals of Operations Research.

[ref-36] Paparrizos K, Samaras N, Stephanides G (2003a). An efficient simplex type algorithm for sparse and dense linear programs. European Journal of Operational Research.

[ref-37] Paparrizos K, Samaras N, Stephanides G (2003b). A new efficient primal dual simplex algorithm. Computers and Operations Research.

[ref-38] Paparrizos K, Samaras N, Triantafyllidis C (2008). A computational study of exterior point simplex algorithm variations.

[ref-39] Paparrizos K, Samaras N, Tsiplidis K, Floudas CA, Pardalos PM (2001). Pivoting algorithms for linear programming generating two paths. Encyclopedia of optimization.

[ref-40] Samaras N (2001). Computational improvements and efficient implementation of two path pivoting algorithms. PhD thesis.

[ref-41] Terlaky T (1985). A convergent criss-cross method. Optimization.

[ref-42] Terlaky T, Zhang S (1993). Pivot rules for linear programming: a survey on recent theoretical developments. Annals of Operations Research.

[ref-43] Triantafyllidis C, Samaras N (2014). Three nearly scaling-invariant versions of an exterior point algorithm for Linear Programming. Optimization: a Journal of Mathematical Programming and Operations Research.

[ref-44] Triantafyllidis CP, Papageorgiou LG (2018). An integrated platform for intuitive mathematical programming modeling using LaTeX. PeerJ Computer Science.

[ref-45] Yang L, Liu S, Tsoka S, Papageorgiou LG (2016). Mathematical programming for piecewise linear regression analysis. Expert Systems with Applications.

[ref-46] Yeh WC, Corley HW (2009). A simple direct cosine simplex algorithm. Applied Mathematics and Computation.

[ref-47] Zhang S (1999). New variants of finite criss-cross pivot algorithms for linear programming. European Journal of Operational Research.

